# Cytoplasm protein GFAP magnetic beads construction and application as cell separation target for brain tumors

**DOI:** 10.1186/s12951-020-00729-9

**Published:** 2020-11-18

**Authors:** Yang Zhao, Feng Jiang, Qinhua Wang, Baocheng Wang, Yipeng Han, Jian Yang, Jiajia Wang, Kai Wang, Junping Ao, Xunxiang Guo, Xiaofei Liang, Jie Ma

**Affiliations:** 1 Department of Pediatric Neurosurgery, Shanghai Xin Hua Hospital Affiliated To Shanghai Jiaotong University, School of Medicine, No. 1665 Kongjiang Road, Shanghai, 200092 China; 2 State Key Laboratory of Oncogenes and Related Genes, Shanghai Cancer Institute, Renji Hospital, Shanghai Jiaotong University School of Medicine, No. 25/Ln 2200 Xie Tu Road, Shanghai, 200032 China; 3 Key Laboratory of Systems Biomedicine (Ministry of Education), Shanghai Center for Systems Biomedicine, Shanghai Jiao Tong University, Shanghai, 200240 China

**Keywords:** Circulating tumor cell, Cytoplasm protein, Liquid biopsy, Tumor diagnosis, Brain tumor

## Abstract

**Background:**

It is very important to develop a highly efficient cerebrospinal fluid (CSF) detection system with diagnosis and prediction function, for which the detection of circulating tumor cells (CTCs) in CSF is a good choice. In contrast to the past use of epithelial EpCAM as CTCs separation target, a cytoplasm protein of GFAP antibody was first selected to construct highly-sensitive immunomagnetic liposome beads (IMLs). The validation and efficiency of this system in capturing CTCs for brain tumors were measured both in vitro and in vivo. The associations between the numbers of CTCs in patients with their clinical characteristics were further analyzed.

**Results:**

Our data show that CTCs can be successfully isolated from CSF and blood samples from 32 children with brain tumors. The numbers of CTCs in CSF were significantly higher than those in blood. The level of CTCs in CSF was related to the type and location of the tumor rather than its stage. The higher the CTCs number is, the more possibly the patient will suffer from poor prognosis. Genetic testing in GFAP CTC-DNA by sanger sequencing, q-PCR and NGS methods indicated that the isolated CTCs (GFAP+/EGFR+) are the related tumor cell. For example, the high expression of NPR3 gene in CSF CTCs was consistent with that of tumor tissue.

**Conclusions:**

The results indicated that GFAP-IML CTCs isolation system, combined with an EGFR immunofluorescence assay of antitumor marker, can serve as a brand-new method for the identification of CTCs for brain tumors. Via lumbar puncture, a minimally invasive procedure, this technique may play a significant role in the clinical diagnosis and drug evaluation of brain tumors.
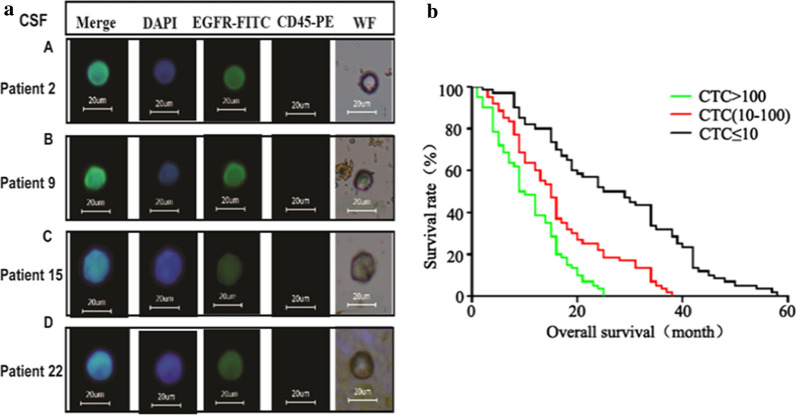

## Background

Liquid biopsy is considered as a promising technique to decipher the characteristics of malignant tumors [1, 2]. Apart from blood testing, it can be extended to monitor other body fluids, such as cerebrospinal fluid (CSF) for tumors in the central nervous system (CNS) [3] including astrocytoma, ependymoma, medulloblastoma and so on. Diverse clinical manifestations with poor subjective and objective descriptions of symptoms often make the diagnosis difficult and lead to misdiagnosis [4]. Therefore, the clinical assessment of brain tumors mainly relies on radiological examinations, such as CT and MRI [5–7]. Nevertheless, factors such as radiation necrosis, hemorrhage, and inflammation may vastly affect radiologists, preventing a correct judgment [8]. In addition, the lack of circulating serum markers limits valuable methods for early clinical assessment and reduces the available options for monitoring disease progression.

Circulating tumor cells (CTCs) refer to tumor cells that are found in the peripheral circulation or other body fluids and are derived or detached from primary tumor sites [9]. As a promising liquid biopsy technique, detecting and analyzing CTCs is an ideal way of identifying the nature of tumors with a minimally invasive approach [10]. In the past 5 years, CTCs detection has been widely used to diagnose and assess tumor progression in various malignant tumors, such as breast cancer [11], lung cancer [12], liver cancer [13], prostate carcinoma [14] and, recently, brain tumors [15]. The conventional CTCs positive sorting method mainly involves a positive enrichment method designed on the basis of high expression markers on the surface of tumor cells such as EpCAM labeled on the surface of immunomagnetic spheres or microfluidic chips [16, 17]. However, the positive sorting method based on CTCs surface markers (EpCAM, EGFR and other surface markers) cannot obtain more representative tumor cells, resulting in missed selection of many cells with different molecular phenotypes, which has severely restricted the CTCs application value [18, 19]. Can cytoplasmic proteins be selected as an effective target for CTCs separation, especially in tumors with unreliable surface markers? In this study, for the first time, we introduced the endocytic GFAP antibody of neuroepithelial tumors (NT) [20, 21] as a positive CTCs sorting marker to verify its feasibility in the diagnosis of brain tumors in children.

Due to the existence of a blood–brain barrier in the central nervous system, the detection of CTCs in CSF was thought to be much more efficient than that in peripheral blood [22]. Recent studies on brain metastasis of epithelial tumors such as breast and lung cancer have demonstrated the successful isolation and identification of CTCs from CSF of patients [23–25]. However, the detection and application of CTCs for CNS brain tumor were unclear and had very few reports [26]. Herein, with a view to preliminary clinical validation, the cytoplasmic protein GFAP was selected to construct an immunomagnetic liposome, which was used as a CTCs isolation system for NTs.

## Results

### Construction and evaluation of GFAP-IMLs

**Fig. 1:**
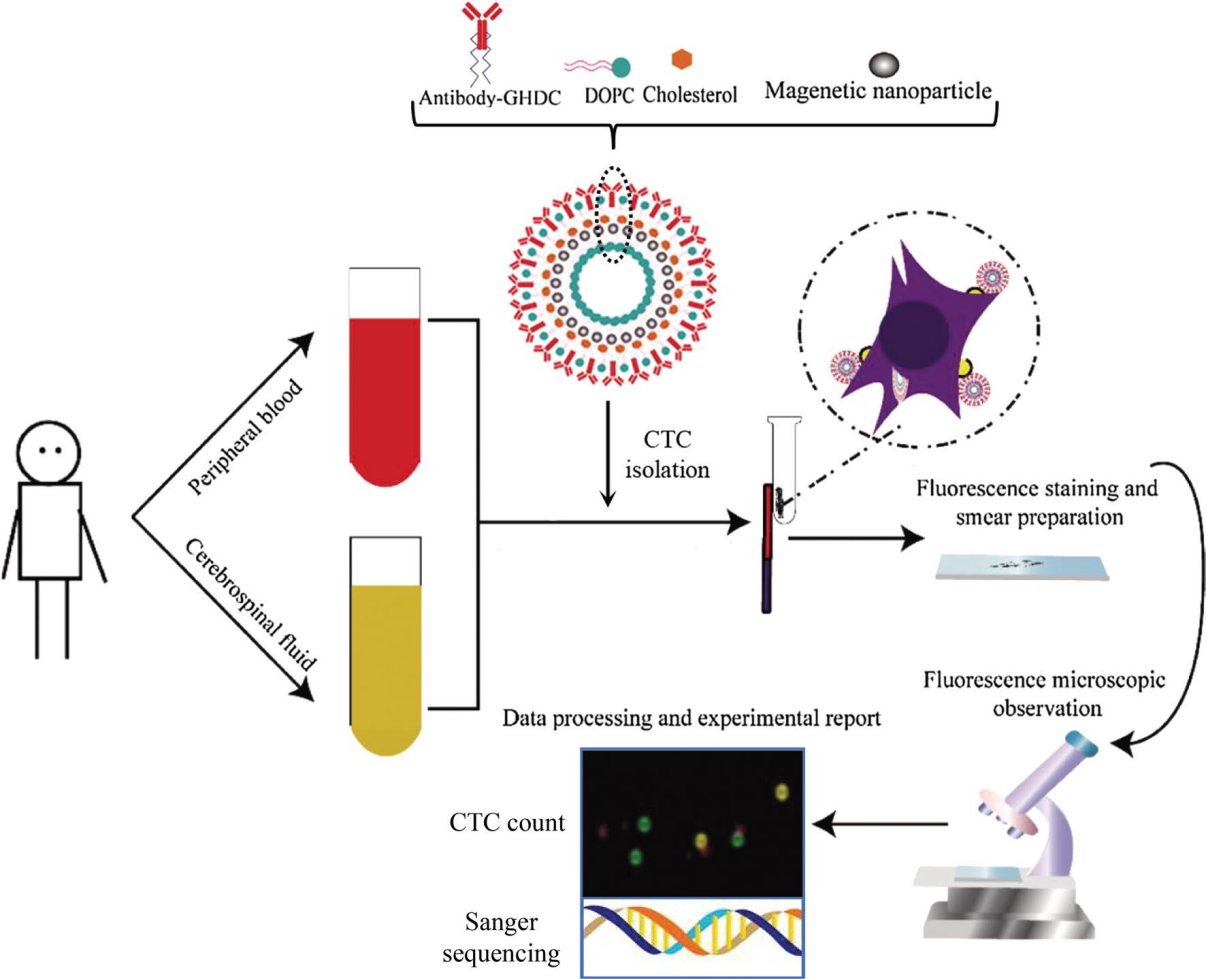
Preparation process of the IMLs and the flow chart of CTCs isolation and identification by IMLs in children with brain tumors in this study

This study was to construct CNS neoplasms CTCs separation system based on GFAP antibody immunolipid magnetic liposomes and to verify it in brain tumors. GFAP-IMLs were prepared by reverse emulsification method by using GFAP-GHDC, DOPC and cholesterol. Fe_3_O_4_ nanoparticles were wrapped in liposomes, and the long chain alkyl part of amphiphilic GFAP-GHDC was inserted into the lipid bilayer membrane, and most of the GFAP antibodies were distributed on the surface of the liposomes. After CTCs separation of collected CSF or blood was performed by GFAP-IMLs, DAPI/EGFR-FITC combination immunofluorescence staining was used for identification and CTCs counting. Meanwhile, DNA extraction and gene testing of selected CTCs groups could be performed (Fig. 1).

**Fig. 2:**
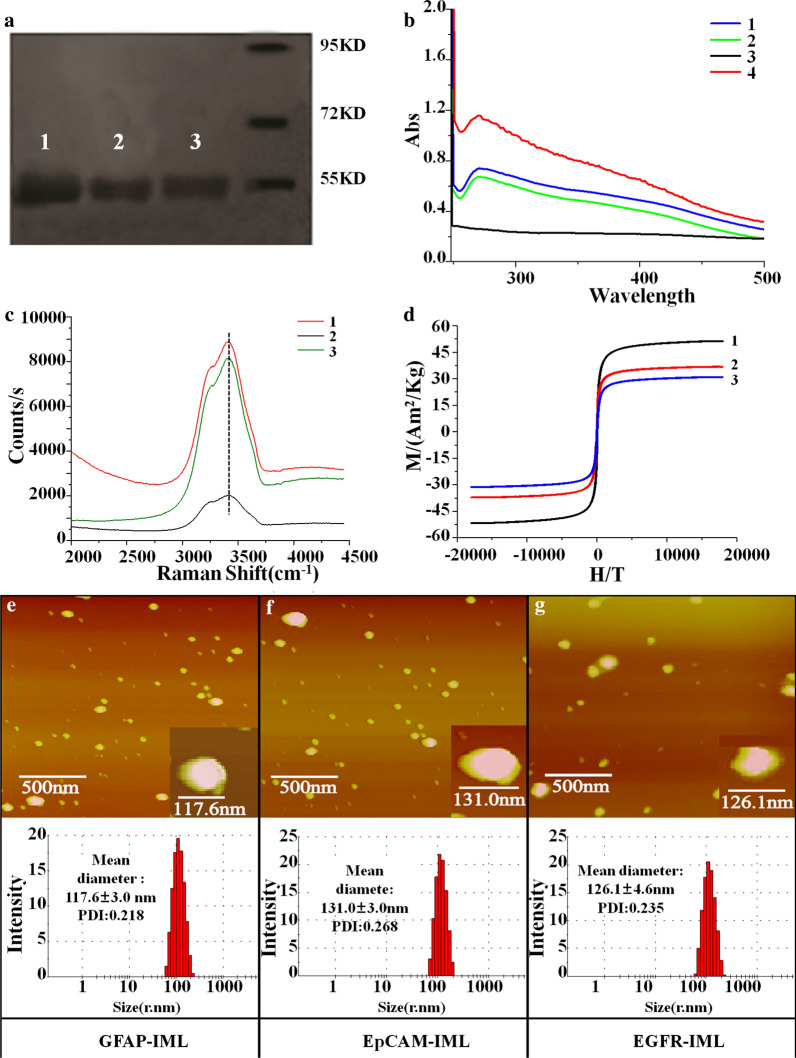
Material characteristics of the three IMLs. **a** Protein electrophoresis. Lanes 1–3 were GFAP, GFAP-GHDC, and GFAP-IMLs, respectively; **b** UV spectra. Lines 1–4 indicate the GFAP, GFAP-GHDC, Fe_3_O_4_ raw magnetic beads and GFAP-IMLs; **c** Raman spectra. Lines 1–3 indicate the GFAP, GFAP-GHDC, and GFAP-IMLs; **d** VSM magnetization curves. Lines 1–3 demonstrate the Fe_3_O_4_ raw magnetic beads, magnetic liposomes, and antibody-IMLs, respectively. **e** Upper: AFM topographic image and below: particle size distribution of GFAP-IMLs; **f** Upper: AFM topographic image and below: particle size distribution of EpCAM-IMLs; **g** Upper: AFM topographic image and below: particle size distribution of EGFR-IMLs

The microstructure and physicochemical characterization of GFAP-IMLs were analyzed and compared with EpCAM-IMLs. Clear protein bands were observed in GFAP-GHDC and GFAP-IMLs in protein electrophoresis photo, indicating that the GFAP antibody was successfully inserted into the lipid bilayer of IMLs (Fig. 2a). The UV absorption spectrum of IMLs showed that the GFAP antibody, GFAP-GHDC and GFAP-IMLs had clear UV absorption peaks near 280 nm. However, with the modification of antibodies and the influence of nanospheres on UV absorption, the absorption peaks of the antibody derivatives and IMLs at 280 nm become weaker, wider and slightly shifted (Fig. 2b). The molecular configurations of GFAP antibody, GFAP-GHDC and GFAP-IMLs were studied by in situ electrochemical Raman spectroscopy. Raman spectra revealed the same characteristic peaks for all samples (Fig. 2c). The magnetic saturation curve showed that the prepared GFAP-IMLs had a high saturation magnetization, and no hysteresis was detected in the curves of Fe_3_O_4_ raw magnetic beads and GFAP-IMLs (Fig. 2d). The hysteresis curve was closed. The residual magnetic force and coercive force were zero within the allowed range of the instrument, suggesting good superparamagnetic properties. The maximum specific saturation magnetization of magnetofluid Fe_3_O_4_ was 51.3 emu/g and that of GFAP-IMLs was 30.9 emu/g, accounting for only 60.2% of the pure magnetofluid. The EpCAM-IMLs and EGFR-IMLs showed similar magnetic properties.

AFM revealed similar spherical shapes for these three IMLs, with relatively low distribution uniformity sizes (Fig. 2e–g). The particles size distribution is within the range of 10~250 nm in diameter, and the surface of the beads was coarse. When the particle image was further enlarged (lower right corner), it was revealed that the shapes of these IMLs were irregular, and the magnetic bead surfaces had an antibody lipid membrane. The particle size and zeta potential of the three IMLs were not significantly different, as presented in the respective AFM images. TEM image showed that the GFAP-IML particles size was not uniformly dispersed, including small balls less than 20 nm or large balls more than 100 nm. Small magnetic spheres tend to aggregate into large ones (Additional file 1: Fig. S1). A smaller particle size was more beneficial to the interaction between the beads and cells to improve the separation efficiency of CTCs.

As indicated in the cytotoxicity assay, the inhibition rate of the three IMLs constructed in this study on glioma cells was low at a concentration of < 100 µg/ml, and the cytotoxicity in glioma cells increased gradually as the concentration rose higher than 200 µg/ml. These results also suggested an equal inhibitory effect of the three IMLs on tumor cells, and the inhibitory ability was only related to the concentration of the IMLs added.

The cell inhibition rate of GFAP-IMLs was investigated by using U251 (Additional file 1: Fig. S2) and D425 (Additional file 1: Fig. S3) cells, respectively. The results showed that GFAP-IMLs had no significant inhibitory effect on the proliferation of the two cells for 0.5 h compared with untreated cells. With the extension of the action time, the cells were inhibited to a certain extent, but the survival rates were still more than 80% within 24 h. Therefore, in the cell sorting experiment, we kept the interaction time between GFAP-IMLs and target cells within 0.5 h.

**Fig. 3:**
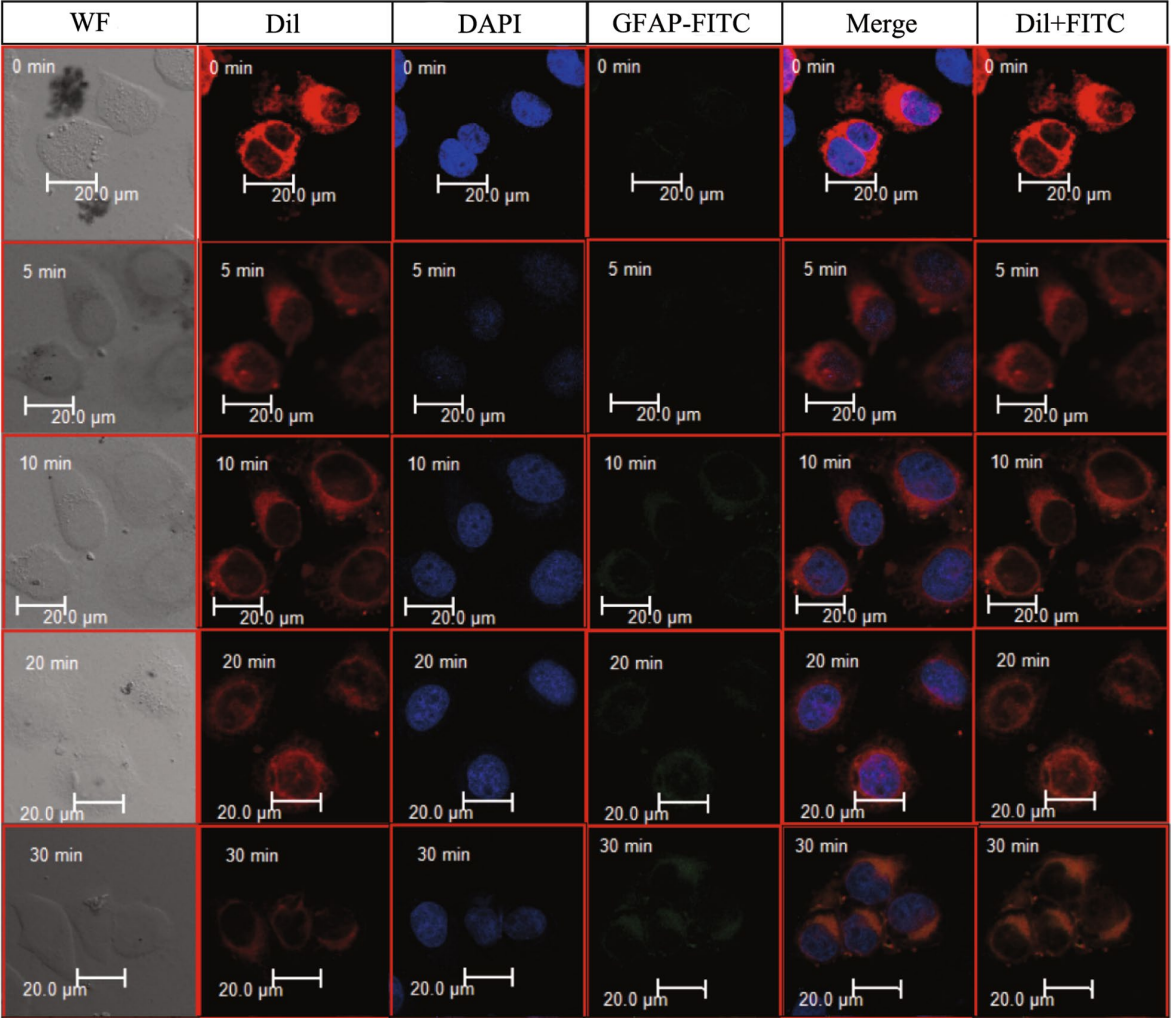
Adhesion and internalization of GFAP-IMLs in U87 cells. Upper to lower panels represent GFAP-IMLs labeled by FITC at different time points (0–30 min) in U87 cell plasma, Scale bar: 20 μm

The combination process between GFAP-ILs and U87 cells treated with fluorescent dye were observed by laser scanning confocal microscope (LSCM) (Fig. 3). The nucleus was shown with DAPI blue fluorescence and the cell surface has DiI red fluorescence. DiI is a lipophilic membrane dye. The red fluorescence clearly shows the outline of the cell membrane. The fluorescence intensity of U87 cells increased gradually over time, indicating an increase of the amount of GFAP-IMLs labeled by FITC. The bright-field view demonstrated the reduction of GFAP-IMLs outside the cells. A large number of GFAP-ILs were adhered on the cell surface at 10 min. The penetration of GFAP-IMLs into the cells significantly increased after 20 min. Therefore, to separate the target cells, the incubation time of GFAP-IMLs could be limited in 30 min. Magnetic separation with a higher cell recovery rate was conducted in 20~30 min of incubation in the following experiment. It can be seen that the GFAP-IMLs prepared in this study have a good affinity with U87 cells. Furthermore, the FITC-GFAP-IMLs uptake was compared between U87 cells with high GFAP expression and JEG-3 cells without GFAP expression in order to demonstrate the specificity of GFAP-IMLs to U87 cells. The interaction time of GFAP-IMLs with cells was 25 min. The results showed that GFAP-IMLs could specifically bind U87 cells and enter the cells to present green fluorescence. However, the surface and interior of JEG-3 cells showed no significant fluorescence (Additional file 1: Fig. S4).

In order to further confirm the interaction between GFAP-IML and U87 cells, we selected GFP-labeled U87 cells and prepared R123-labeled GFAP-IML. Spontaneous signals of GFP in U87-GFP cells were observed from 0 to 30 min through immunofluorescence. At 5 min of incubation time, R123 could not be observed. However, with the extension of incubation time, the red fluorescence became visible and gradually enhanced. At 15 min, the R123 fluorescence was not strong enough, but the cell contour could be seen, indicating that the R123-GFAP-IMLs began to enter the cell interior. The entire cell contour was clearly visible at 25–30 min, suggesting that the R123-GFAP-IMLs had accumulated inside the U87 cells (Additional file 1: Fig. S5).

**Fig. 4:**
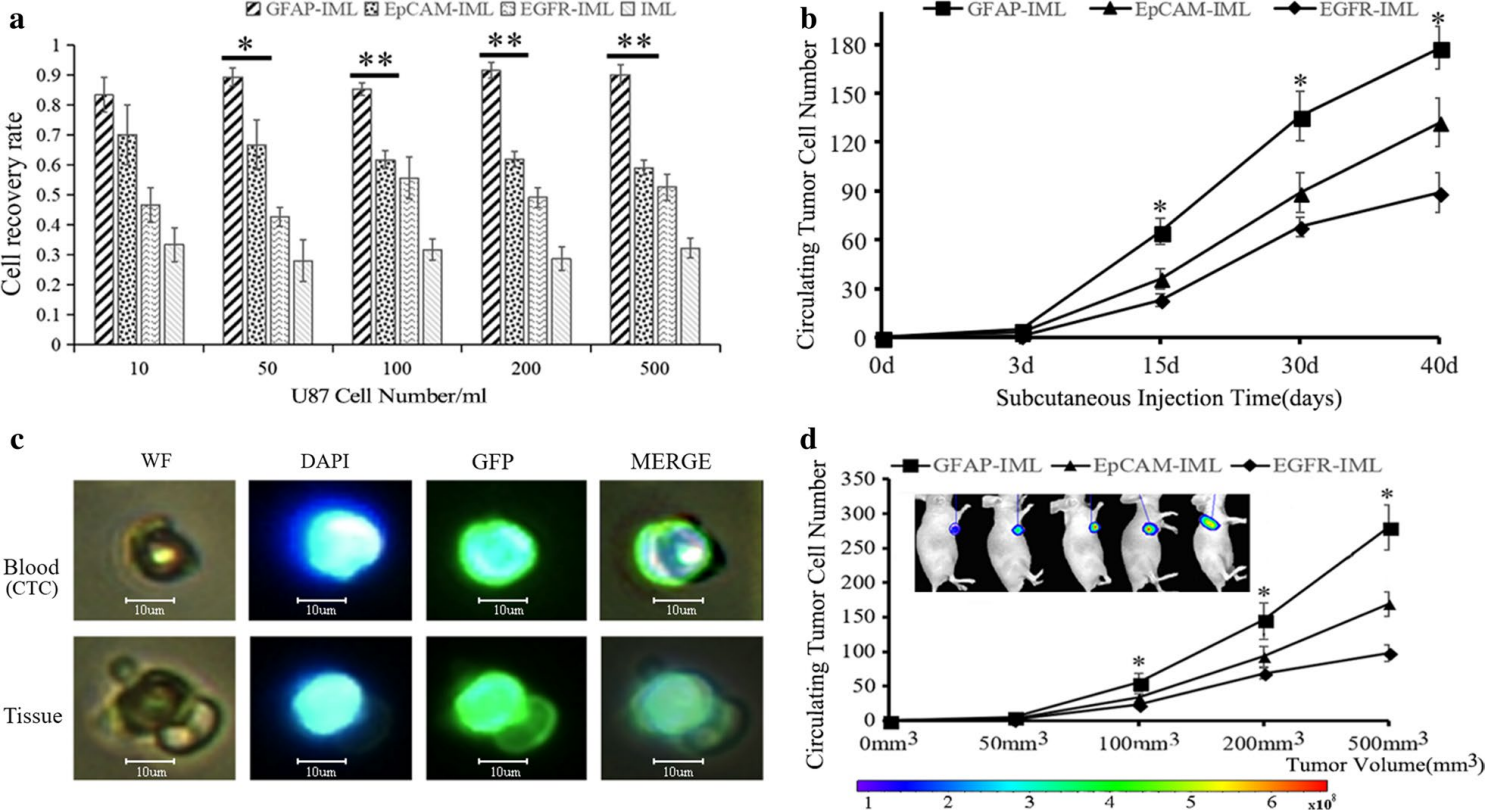
Verification of the utility of IMLs in the isolation and identification of CTCs in vitro and in vivo. **a** Comparison of the cell capture efficiency among the three IMLs in PBS; **b** The number of CTCs isolated by three IMLs from mice at different time points after subcutaneous injection (*P < 0.05, **P < 0.01); C: Validation of CTCs captured by IMLs from blood and tumors in nude mice; D: The numbers of CTCs isolated by three IMLs from tumors with different volumes (*P < 0.05, **P < 0.01)

The results showed that three IMLs were able to capture U87 cells suspended in PBS under different concentration gradients. At the same antibody content on IMLs with the same magnetic quality, the average efficiency of GFAP-IMLs, EpCAM-IMLs, EGFR-IMLs and IMLs in capturing U87 was 87.9%, 63.8%, 49.4% and 30.8%, respectively. The capture efficiency of GFAP-IMLs in PBS was higher than those of EGFR-IMLs and EpCAM-IMLs (Fig. 4a). Children medulloblastoma D425 was also selected to evaluate the cell separation efficiency for three IMLs. The results showed that the average separation efficiency of GFAP-IMLs, EpCAM-IMLs, EGFR-IMLs and IMLs was 92.4%, 25.8%, 91.2% and 17%, respectively (Additional file 1: Fig. S6). It also indicated that D425 was highly expressed for GFAP and EGFR. There was no significant difference in the separation efficiency between GFAP-IMLs and EGFR-IMLs, which indicated that cytoplasmic protein GFAP can be selected as medulloblastoma cell separation target.

U87 cells were subcutaneously injected into 4-weeks-old nude mice. Three kinds of IMLs were used to mimic the capture of CTCs in the blood from nude mice after 3, 15, 30 and 40 days of subcutaneous injection. All IMLs could capture CTCs in the blood, and the number of CTCs captured by GFAP-IMLs was significantly higher than those captured by EGFR-IMLs and EpCAM-IMLs (Fig. 4b). Fluorescence images also showed that CTCs captured by GFAP-IMLs from the blood and tumor tissues of nude mice were similar in cell morphology to U87-GFP cells with spontaneous green fluorescence (Fig. 4c). The numbers of CTCs captured were positively correlated with the sizes of inoculated tumors, which were 0 mm^3^, 50 mm^3^, 100 mm^3^, and 200 mm^3^ in nude mice. In addition, when the tumor volume exceeded 50 mm^3^, the numbers of CTCs isolated by GFAP-IMLs were significantly higher than those isolated by the other two IMLs (*p* < *0.05*). Through in vivo imaging of nude mice, it was confirmed that the tumor was composed of U87 cells with GFP green fluorescence (Fig. 4d).

### Preliminary clinical application of IMLs in brain tumors

**Fig. 5:**
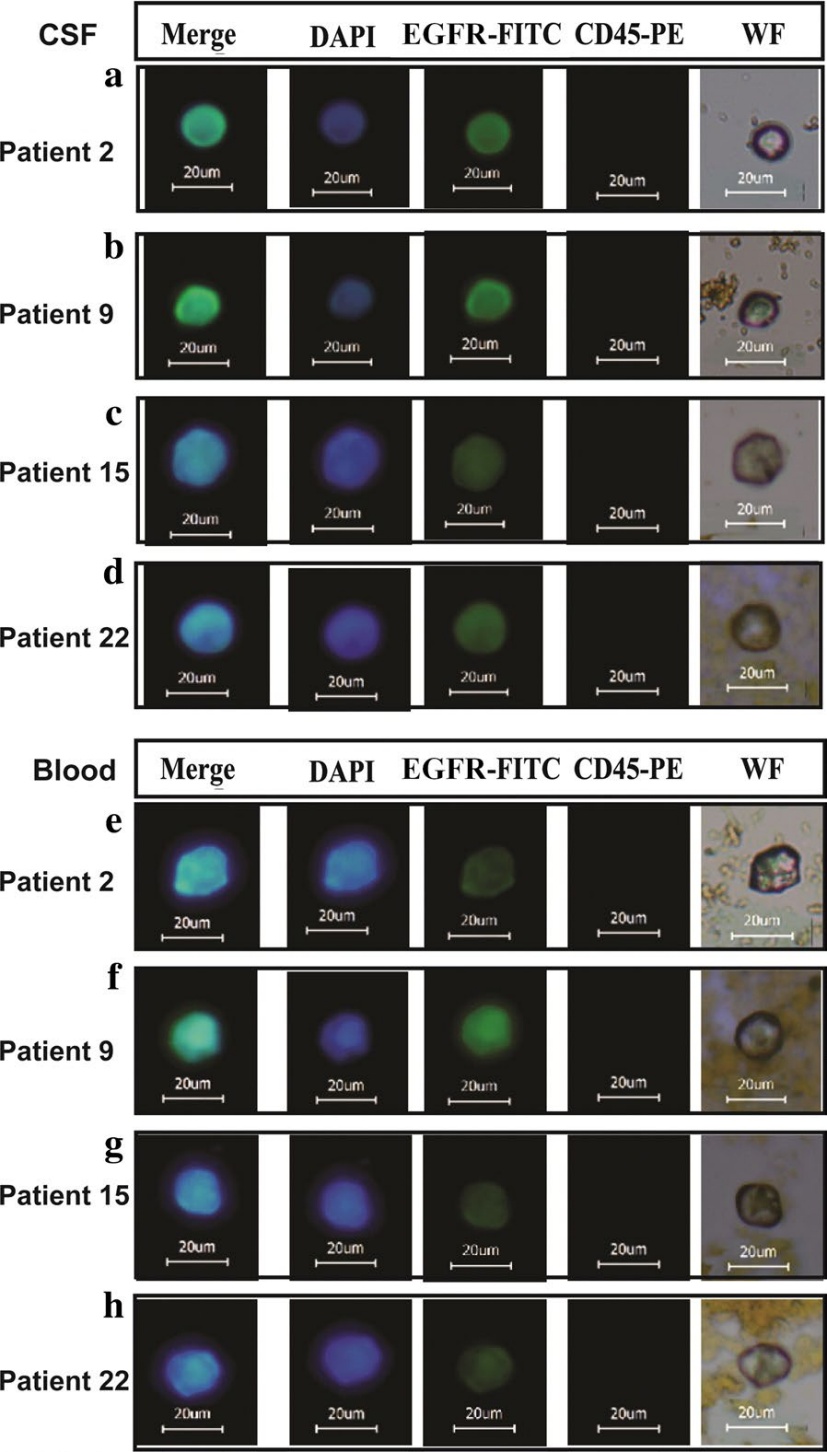
Validation of three IMLs in the isolation of CTCs from patients with pediatric brain tumors. (A-D): Representative fluorescence in cells isolated by GFAP-IMLs from patients’ peripheral blood. Isolated cells were labeled with an anti-GFAP monoclonal antibody conjugate by FITC (scale bar: 20 μm); (E–H): Representative fluorescence in cells isolated by GFAP-IMLs from patients’ CSF. Isolated cells were labeled with an anti-GFAP monoclonal antibody conjugate by FITC (scale bar: 20 μm)

Thirty-two children (3 abnormal data points, which were much higher than the average, were deleted) with brain tumors were recruited in this study (Additional file 1: Table S1). GFAP-IMLs were applied to capture CTCs in both peripheral blood and CSF from these patients. EpCAM and EGFR IMLs were used as the control group. As a verification approach, captured CTCs were labeled by anti-EGFR antibody conjugated FITC and anti-CD45 antibody conjugated by PE. The patients’ CTCs images with GFAP high expression isolated from blood and CSF are shown (Fig. 5). All cells are 15~20 μm in size, with strong blue (DAPI) and green (EGFR-FITC) fluorescence. There are also obvious round and oval cell morphology under the white light, and the cell surface is covered with light brown magnetic particles (GFAP-IML nanoparticles).

**Fig. 6:**
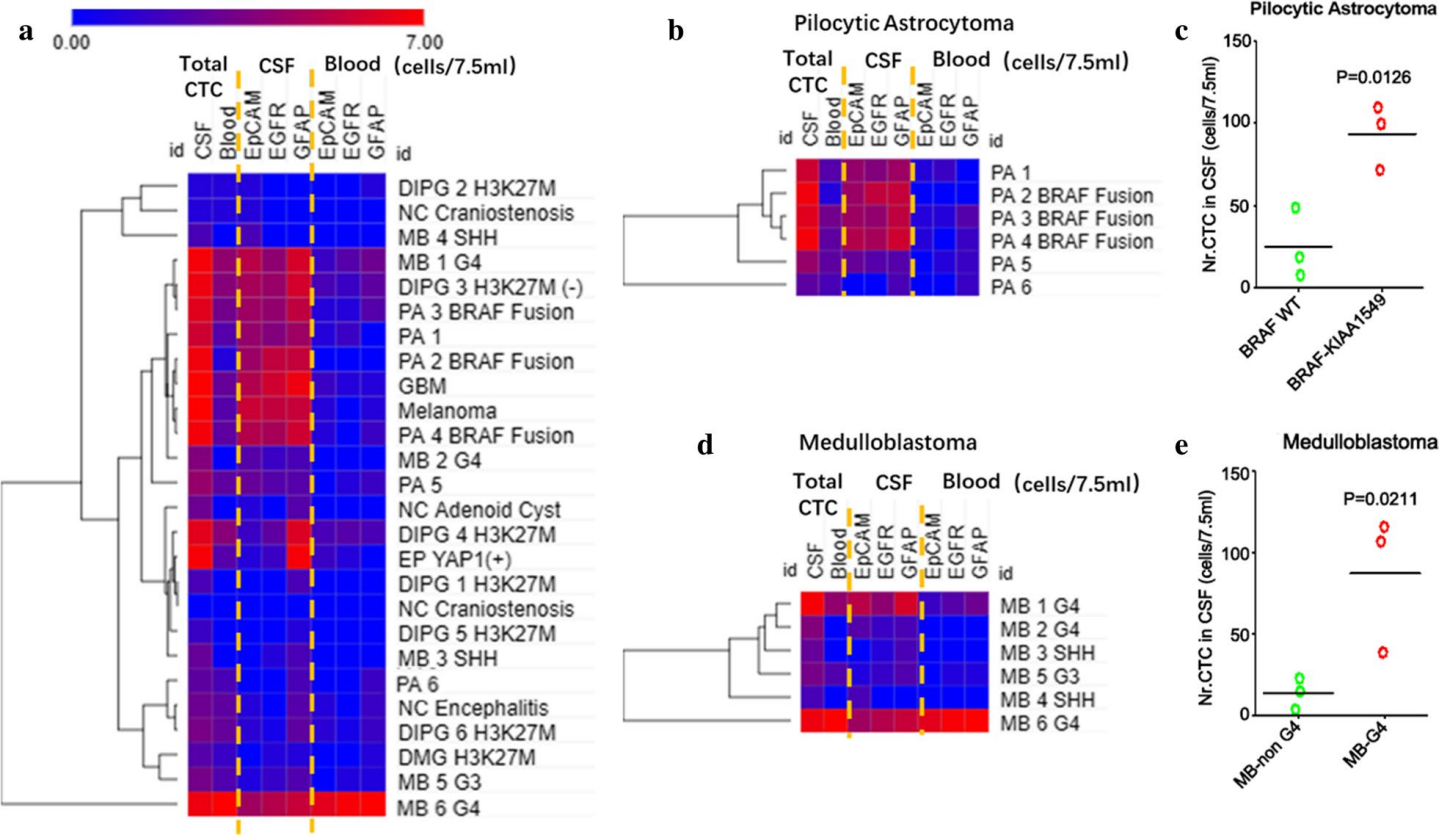
Correlation of isolated CTCs with the clinical information of children with brain tumors. **a** Heat map summarizing isolated CTCs from the peripheral blood and CSF of each patient; **b** Heat map of CTCs isolation from patients with PAs; **c** The dot-plot represents the number of CTCs from the peripheral blood and CSF of each patient with PA; **d** Heat map of CTCs isolation from patients with medulloblastoma; **e** Dot-plot of CTCs isolated from the CSF of patients with medulloblastoma. MB: Medulloblastoma, PA: Pilocytic Astrocytoma, DIPG: Diffuse Intrinsic Pontine Glioma, NC: Normal Control

The numbers of captured cells in peripheral blood and CSF from each sample, together with those in the control group, were calculated and summarized in the scattergram and heat map. The number of CTCs from embryonal tumors was lower overall than any other types of tumor (Fig. 6a). For other brain tumors, it was interesting to find that in pilocytic astrocytoma (PA) cases with KIAA1549-BRAF fusions, more CTCs were recruited than those in wild-type cases. Additionally, more CTCs could be captured in CSF samples than in peripheral blood samples from PA patients (Fig. 6b, c). These results indicated that the CTCs separation system might be a minimally invasive procedure for diagnosing children with images revealing suspected PA cases. Furthermore, this system may provide a novel method for posttreatment assessment of efficacy. In medulloblastoma, group 4 cases tended to have more CTCs than non-group 4 cases in both CSF and peripheral blood samples (Fig. 6d, e). This finding indicated that group 4 medulloblastoma had a likely higher frequency of metastatic. In addition, one patient exhibiting cerebellar glioblastoma with CSF dissemination also showed a higher CTCs number (Additional file 1: Fig. S7).

**Fig. 7:**
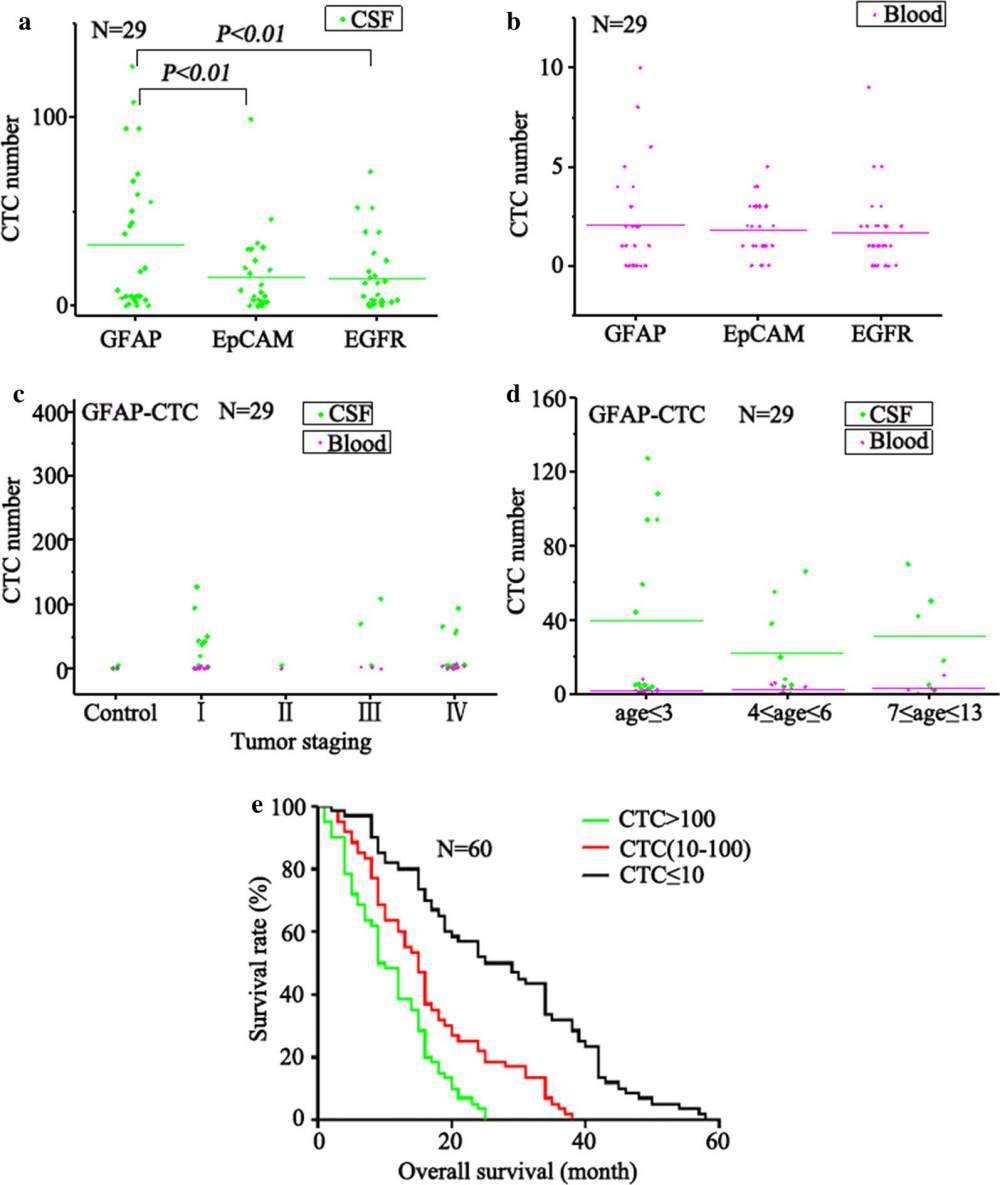
**a** The number of CTCs captured in CSF with the GFAP, EpCAM, and EGFR magnetic spheres. **b** The number of CTCs captured in the blood with the GFAP, EpCAM, and EGFR magnetic spheres. **c** The number of CTCs captured by GFAP magnetic spheres in different tumor stages (CSF and blood). **d** The number of CTCs captured by GFAP magnetic spheres at different ages (CSF and blood). The average number of CTCs is shown by the horizontal lines. **e** The relationship between the amount of CTCs in CSF and survival time

Of course, there was no significant difference in the CTC cells sorted by different immunomagnetic spheres in the blood (Fig. 7a, b). In CSF, the number of CTCs sorted by the GFAP immunomagnetic sphere was significantly higher than that of the EpCAM and EGFR magnetic spheres (*P* < *0.01*). To our surprise, the number of CTCs sorted by GFAP magnetic spheres was not significantly associated with tumor stage, either in cerebrospinal fluid or in blood (Fig. 7c). The number of CTCs sorted by GFAP magnetic spheres was related to the age of the children (Fig. 7d). Children less than 3 years old had more CTCs in their CSF than those in other age groups (*P* < *0.01*). Survival data was available for 60 brain tumor patients (Additional file 1: Table S2), and the median survival analysis was performed for three number cut-off of ≤ 10, 10–100 and ≥ 100 CTCs per 7.5 mL of CSF. The median survival time was 27 months when the number of CTCs was ≤ 10, and the median survival time was 15 months when the number of CTCs was within the range of 10–100. But when the CTCs number was above 100, the median survival was only 9.5 months. There were significant differences among the three CTCs number interval (*P* < *0.001*) (Fig. 7e). It can be deduced that there is a significant correlation between the number of CTCs in CSF and the patient’s survival time. The higher the CTCs number in CSF is, the more possibly the patient will suffer from poor prognosis.

**Fig. 8:**
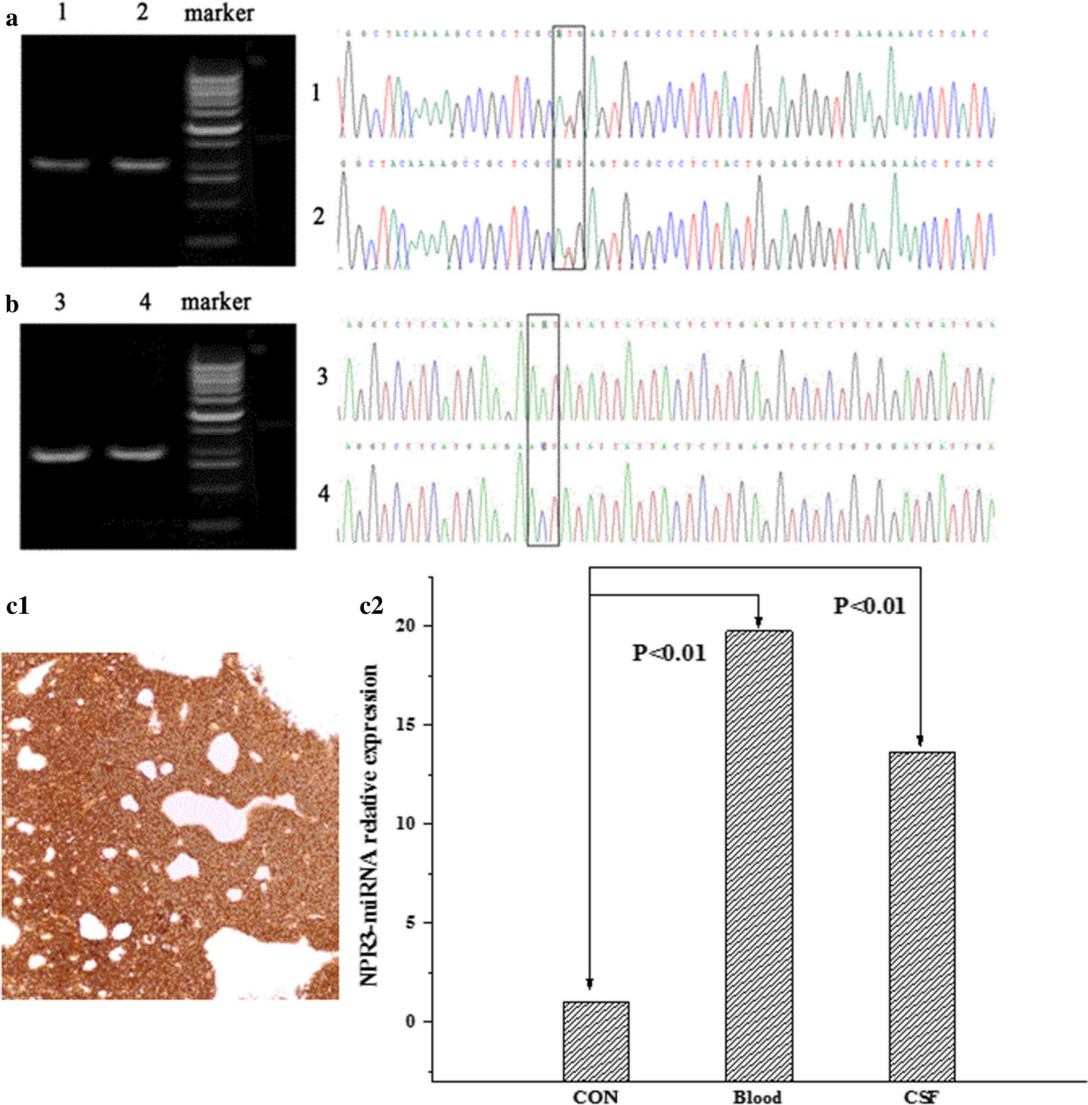
The mutation detection results of H3F3A and BRAF genes in DIPG patients. **a** H3F3A gene, including agarose gel electrophoresis and sequencing results. (A1: Tumor tissue; A2: CSF). **b** BRAF gene, including agarose gel electrophoresis and sequencing results. (B3: tumor tissue; B4: blood). **c** The over-expression of NPR3 in CTCs from a patient diagnosed with Group 3 Medulloblastoma by RT-PCR, which is consistent to the result in immunohistochemistry staining of tumor tissue. (C1: IHC staining of NPR3 in tumor tissue, C2: RT-PCR plot)

To strengthen evidences in supporting the origination of isolated cells, distinct genetic alterations were tested in CTCs according to the genetic characteristics in original tumors. We found that the *H3F3A* gene showed *K27M* mutations in CTCs (captured from CSF) and in tumor tissue samples from DIPG patients (Fig. 8). What’s more, CTCs from a patient diagnosed with medulloblastoma were revealed with over-expressed NPR3 by RT-PCR, which was consistent with immunohistochemistry staining of NPR3 protein in tumor tissue (Fig. 8c). What’s more, two same gene mutations of KMT2A and TMPRSS2 were checked out in GFAP separated CTCs and tumor tissue. Cells isolated from the GFAP-IMLs were further verified by whole exon sequencing and compared with the tissue (Additional file 1: Table S3). Blood sorting CTC detected 5 gene mutations and tissue detected 4 gene mutations, all of which were KMT2A and TMPRSS2 mutations, which again demonstrated that the cells sorted by self-made GFAP-IMLs were consistent with the tissues in some genes. In addition, the blood samples detected a code-shifting mutation in exon 20 of CIC gene, with a richness of 9.62%, which may lead to truncation mutation of the protein, thus affecting the protein function. Five RNA sequencing data isolated by GFAP-IMLs were analyzed (Additional file 1: Fig. S8). The detection results were screened according to the difference significance criteria (the difference in gene expression was more than 2 times and q ≤ 0.05), and the down-regulation of the significant difference in gene expression was counted. A total of 2551 RNA expressions with significant differences were screened out, among which 1238 were up-regulated and 1313 were down-regulated.

## Discussion

In this study, GFAP antibody combined with the lipid material GHDC and DOPC was selected as the matrix of magnetic microspheres to construct GFAP-IMLs for CTCs separation, with low cytotoxicity and high biocompatibility. It is stable with controllable surface antibody content, and has the crystallization properties of magnetic particles. GFAP is a marker of astrocytes and is widely expressed in most CNS brain tumors [27, 28] Of course, GFAP was expressed in 85.7% of NT and higher GFAP positivity was found in glioma than other types of NT (P value < 0.05) [20, 29]. Herein, we for the first time attempted to select GFAP as the main target of positive CTCs sorting, and to investigate its efficiency as a CTCs sorting agent in CSF of brain tumors.

These in vitro and in vivo tests indicated that the GFAP-IMLs were more specific in capturing U251, U87 and D425 cells. In summary, through blood CTCs capture in vivo experiment, we confirmed that CTCs could be released into the peripheral blood during the process of tumor development and volume enlargement. Different from the reference of CTC separation method by microfluidic technology for GBM [26], antigen-dependent positive CTC selection method by GFAP target is effective. Similar to the literature, CTCs can be observed by cytospin collection with GFAP positive and the presence of EGFR amplification [26, 30, 31]. GFAP-IMLs separation combined with EGFR fluorescent identification system in the present study could more efficiently isolate GFAP positive CTCs both in vitro and in vivo contexts, which demonstrated the necessity of clinical tests.

The preliminary clinical application of GFAP-IMLs for CTCs separation was verified by thirty-two children with different brain tumors (Table S1). In general, the numbers of CTCs in CSF were significantly higher than those in peripheral blood, and the numbers of CTCs in tumor patients were significantly higher than those in normal control individuals. Similar to the CTC (EpCAM+/CK+) sorting in the blood of lung cancer patients develop brain metastases often reported in the literature, GFAP has a greater advantage in the sorting of brain tumor cells in CSF [32]. Above number of 300 CTCs can be found in CSF in anaplastic ependymoma patients, while only bare CTCs can be isolated in blood. These results have indicated that cytoplasm GFAP can be selected as an effective CTCs separation protein. The number of CTCs sorted by GFAP-IMLs was related to the age of children, but not associated with tumor stage. We also found that there is a correlation between the number of CTCs in CSF and the patient’s prognosis. The higher the CTCs number is, the more possibly the patient will suffer from poor prognosis.

Furthermore, gene mutation detection in separated CTCs by GFAP-IMLs also indicated the tumor-derived circulating cells. Through whole-genome sequencing of DNAs from seven diffuse intrinsic pontine gliomas (DIPGs), 78% of DIPGs contained *H3F3A* mutations, which was consistent with a previous report [33]. In addition, nearly 80% of DIPGs had the lysine-to-methionine mutation, the famous H3K27M [33, 34], and PCR and sanger sequencing were used to perform genetic testing on one portion of DIPG patients. *H3F3A* gene mutations in CSF CTCs and in tumor tissue sample were detected from one of the DIPG patients. What’s more, NPR3 overexpression is a characteristic phenomenon in identification of Group 3 Medulloblastoma [35]. The detection of over-expressed NPR3 in the CTCs from a Group 3 Medulloblastoma was another evidence in supporting the specificity of CTCs isolated by IMLs. NGS method in CTC-DNA detection also indicated that the GFAP targeted CTCs has the features of brain tumors. RNA-seq results indicated that there were significant differences in RNA expression between the 5 groups isolated by GFAP-IMLs. It can also be seen that the expression of genes is also clustered, and each gene group has similar expression levels. Indeed, large sample validation still needs to be carried out in our further study. Our findings point towards a possibly biological pattern of CTC dissemination in patients with brain tumor.

## Conclusions

In summary, the cytoplasm protein GFAP nanoparticles for magnetic separation prepared in the present study effectively isolated CTCs in CSF and peripheral blood from children with brain tumors. The constructed GFAP-IMLs separation system with DAPI/EGFR-FITC immunofluorescence assay was effective in the detection of CTCs from CSF and peripheral blood samples. Consequently, this system might be a novel approach providing a valuable reference in the early diagnosis, preoperative and postoperative analysis in brain tumors and other tumors with cytoplasm protein high expression.

## Methods

### Preparation of GFAP immunomagnetic liposome beads (GFAP-IMLs)

GFAP-IMLs were prepared by using the reverse-phase evaporation (REV) method. Briefly, 5 mg dioleoylphosphocholine (DOPC) (Avanti Company) and 5 mg cholesterol were obtained and added into a 50 ml 3-neck flask. After removing ethanol, 1.0 mg Fe_3_O_4_-hydrophobic magnetic nanoparticles (HMNs) were dissolved in 3.0 ml CH_2_Cl_2_ and transferred into the previously prepared 3-neck flask. The mixture in the flask was sonicated on ice. Simultaneously, 2 mg GFAP antibody (Abcam, ab7260) modified glycidyl hexadecyl dimethylammonium chloride (GFAP-GHDC) was dissolved in 6 ml ddH_2_O and gradually added to this flask. The preparation method of GFAP-GHDC refers to our previous method [36]. After emulsification, rotary evaporation was used to remove the residual CH_2_Cl_2_ from the emulsion. The solution was magnetically separated and washed three times, and GFAP-IMLs were obtained. Similarly, EpCAM (Abcam, ab71916) modified IMLs were constructed. The detailed preparation process and reagent consumption are described in our previous study [37].

### Characterization of IML

Zetasizer Nano-ZS 90 (Malvern Instruments, Ltd., UK) was used to determine the particle size and potential of IMLs. Atomic force microscopy (AFM) was used for the micromorphology of different IMLs. Magnetic hysteresis loops of these magnetic particles were detected using PPMS-9 (QUANTUM DESIGN, USA). An ultraviolet spectrophotometer was used to confirm the presence of antibodies on the surface of IMLs and to analyze the antibody content qualitatively. A bicinchoninic acid assay (BCA assay) was used to quantitatively analyze the antibody content of these IMLs. Polyacrylamide gel electrophoresis (PAGE) was used to detect the antibody content and to confirm the presence of the antibodies on the surface of these IMLs.

### Cytotoxicity assay of the IMLs

U87, U251 and D425 Cells were suspended and seeded into a 96-well plate with 8,000 cells in each well containing 100 µl culture medium and cultured overnight. IMLs were added into each well at a final concentration gradient of 0, 10, 50, 100, 200, 500, and 1000 µg/ml. After culturing at 37 °C for 48 h, 10 µl of 5 mg/ml MTT reagent was added to each well. After 3 h of incubation, 150 µl DMSO was added to each well to dissolve the crystal after discarding the medium, followed by measuring at 490 nm using a spectrometer (SpectraMax M5/M5e).

### Evaluation of the interaction between IMLs and brain tumor cells

Commercial glioma cells U87, U251 and D425 were routinely cultured in DMEM complete culture medium supplemented with 10% FBS and 1% penicillin–streptomycin in a humidified 5% CO_2_ incubator at 37 °C. The cytotoxicity assay of IMLs is described in the Additional file. Sterile slides were placed in a 24-well plate, and 1 × 10^4^ U87 cells were seeded into each well containing 1 ml culture medium. These cells were cultured in a 5% CO_2_ incubator at 37 °C for 24 h. After refreshment with new medium, IMLs were added to each well at 20 μl/well, and an equal volume of PBS was added to the control wells. After 48 h of incubation, the medium in each well was discarded and washed with PBS. After fixed the cell with paraformaldehyde, 100 μl DAPI staining solution was added to each well and incubated for 5 min. After discarding the staining solution and washing with PBS, cell slides were removed from the wells and placed in an inverted position on a slide-proof glass slide coated with antifade mountant. These slides were observed by a confocal laser scanning microscope (Lcica TCS SP8 STED 3X).

Rhodamine 123 (R123) (Sigma) was encapsulated in the GFAP-IMLs. After dialysis for 5 h to remove the excess free R123, R123-labeled GFAP-IMLs were mixed with U87-GFP cells and incubated at 37 °C for different minutes. The final dyeing effect of U87-GFP cells was observed under a fluorescence microscope after washing with PBS.

### Experiment on the recovery rate of simulated CTCs by IML

Different (10–200) suspended U87 cells were mixed with 7.5 ml PBS with anticoagulant to mimic the CTCs in CSF and blood. The abilities of GFAP-IMLs, EpCAM-IMLs, EGFR-IMLs and IMLs to capture CTCs were measured. The cell suspension was dropped onto a slide-proof polylysine-coated glass slide, which was subjected to a fluorescence microscope after drying out. The experimental procedure of CTCs detection is the same as that in our previous study [36] and described in the following of the experimental procedure of CTCs detection**.**

### U87-GFP cell separation efficiency of GFAP-IML in vitro and in vivo

U87-GFP cells at the logarithmic growth stage were digested with trypsin to make the cell suspension. A total of 0.2 ml of the prepared U87-GFP cell suspension (1 × 10^7^/ml) was subcutaneously injected into the right back skin of 4-week-old female BALB/c nude mice (Supplied by SLRC Laboratory Animal). Tumor volume was calculated according to the following formula: Tumor volume V (mm^3^) = π/6 × Length (mm) × Width (mm^2^). Measurement was performed once every 2 or 3 days, and the growth rate was calculated according to the following formula: Growth rate(%) = Mean tumor volume (mm^3^)/ Survival time of tumor—Bearing mice (days).

Blood samples (0.1 ml) were collected from nude mice eyeball, from which CTCs were isolated and identified by IML incubation followed by magnetic separation. Similar to the above CTC recovery method, the supernatant was transferred carefully into a 1.5 ml centrifuge tube, and an equal volume of PBS was added and mixed. Then, 30 μl IMLs was added and incubated at RT for 25 min and mixed once every 5 min. Furthermore, 30 μl DAPI, 10 μl EGFR-FITC (Abcam, ab11400) solution, and 10 μl CD45-PE were used to stain isolated cells for 15 min away from light. After washing three times with PBS, the cell suspension was dropped onto a slide-proof glass slide and observed under a fluorescence microscope.

### The experimental procedure of CTCs detection

Each cell sample to be tested was centrifuged at 1000 rpm for 10 min. After centrifugation, the upper layer was carefully removed, leaving the lower layer solution in the tube, and equal volume PBS was added to the tube and mixed well. Then, 20 µl IMLs was added to each tube and incubated for 25 min at room temperature (RT) and mixed every 5 min. The tube was placed on a magnetic separation rack for 10 min, and the IMLs with captured CTCs were washed with PBS twice. DAPI and fluorescent antibody were added and incubated for 15 min away from light to stain the cells, followed by magnetic separation for 5 min at the end of staining. The cells were washed twice with deionized water to fully remove the unbound antibody and DAPI. Finally, 15 µl deionized water was added to the tube to resuspend CTCs. The cell suspension was dropped onto a slide-proof polylysine-coated glass slide for fluorescence microscope inspection.

### Inclusion criteria for patients with brain tumor

The present study was approved by the hospital ethical committee and signed informed consents were obtained from all patients. (1) Children (age ≤ 13) histologically diagnosed with brain tumor by two board certificated pathologists independently; (2) no metastatic lesions with diameter ≥ 1 cm by intracranial MRI; (3) no craniocerebral injury, brain surgery or radiotherapy history within the last 6 months; (4) the symptoms of intracranial hypertension was controlled by dehydration medication; (5) patients could tolerate the lumbar puncture for CSF collection; and (6) patients with intracranial meningiomas, meningioma and meningeal lesions were excluded from the study. 24 patients who met these criteria were enrolled in our study, with 12 males and 12 females.

### Inclusion criteria for patients with non-tumor brain diseases

(1) Patients with non-tumor brain lesions, craniostenosis and scalp mass who were hospitalized in the same period as the experimental group; and (2) patients who needed external ventricular drainage, ventriculoperitoneal shunt, lumbar cistern drainage, lumbar puncture for CSF examination during hospitalization. 3 patients with communicating hydrocephalus met these criteria were included in the control group.

### Detection of CTCs in tumor patients

This study was approved by the Ethics Committee of Xinhua Hospital Affiliated to Shanghai Jiaotong University School of Medicine, and all patients involved in this study signed informed consent. For each patient before tumor resection, 7.5 ml of CSF through lumbar puncture and peripheral blood were collected in an EDTA anticoagulant tube for immediate laboratory examination. Self-prepared anti-GFAP, EpCAM and EGFR IMLs were used to enrich and screen the CTCs in CSF and peripheral blood samples. DAPI was used to identify the intact cells with nuclei, and then cells were stained with fluorescently labeled anti-CD45 and EGFR-FITC (for GFAP-IML)/GFAP-FITC (for EpCAM/EGFR-IML separation) monoclonal antibodies to distinguish the neuroepithelial tumor cells from the white blood cells. CTCs that met the evaluation criteria, i.e., GFAP+, EGFR+, DAPI+ and CD45−, were counted by using a complementary polychromatic fluorescence cell counting instrument.

### DNA extraction and gene detection in CTCs

The CTCs enriched by GFAP-IMLs were used to extract DNA, which was performed according to the instructions of the DNA extraction kit (EZ Bioscience, No. B0007). PCR-related amplification reagents were purchased from Yeasen Biotech (Shanghai) Co., and Ltd. PCR primers were purchased from Sangon Biotech (Shanghai) Co., Ltd. H3F3A, HIST1H3B and HIST1H3C, as well as NPR3 primers were described in our previous literature [30].

H3F3A, HIST1H3B and HIST1H3C, as well as NPR3 primers are as follows:

CATGGCTCGTACAAAGCAGA (H3F3A-F);

GCAAAAAGTTTTCCTGTTATCCA(H3F3A-R);

TTGGGTCCAATAGTTGGTGGT(HIST1H3B-F);

GACTTTTGGTAGCGGCGGAT(HIST1H3B-R);

CGCGCGGGACTTTTGAAATA(HIST1H3C-F);

GCTCGGTGGACTTCTGGTAG(HIST1H3C-R);

CTACGCCTTCTTCAACATTGAG/TAGCTTCAAAGTCGTGTTTGTC(NPR3-F/R).

RNA sequencing steps are as follows. CTC isolated by GFAP-IMLs was collected into the RNase-free centrifuge tube, and RNA extraction kit was used for extraction. After quality verification, library construction and on-machine sequencing were carried out.

The obtained CTC-DNA could be sequenced by fluorescence quantitative PCR, sanger sequencing and whole exome sequencing (WES). The WES steps are as follows. 100 ng CTC-DNA from each sample was extracted for library construction, including pruning and ligation of aptamers. Agilent v2 Human Exon bait kit was used for exon group capture. Illumina HiSeq platform was used to sequence captured DNA samples and integrate paired sequencing readings for each sample.

### Statistical analysis

Data from each group were processed by SPSS 19.0 statistical software and presented by Prism 7.0 software. Measurement data are presented as the mean ± SD and were compared by the rank sum test. A *p* value < 0.05 was considered statistically significant. A heatmap was produced by R 3.3, and the number of CTCs was transferred by log transformation. For other methods employed in this manuscript, please refer to the Additional file 1.

## Supplementary information

**Additional file 1.** Additional figures and tables,

## Data Availability

All data generated and analyzed during this study are included in this published article.

## Abbreviations

CSF: Efficient cerebrospinal fluid
CTCs: Circulating tumor cells
DIPGs: Diffuse intrinsic pontine gliomas
DOPC: Dioleoylphosphocholine
GHDC: Glycidyl hexadecyl dimethylammonium chloride
HMNs: Hydrophobic magnetic nanoparticles
IMLs: Immunomagnetic liposome beads
PA: Pilocytic astrocytoma
