# Correction to: Cytoplasm protein GFAP magnetic beads construction and application as cell separation target for brain tumors

**DOI:** 10.1186/s12951-021-01050-9

**Published:** 2021-10-18

**Authors:** Yang Zhao, Feng Jiang, Qinhua Wang, Baocheng Wang, Yipeng Han, Jian Yang, Jiajia Wang, Kai Wang, Junping Ao, Xunxiang Guo, Xiaofei Liang, Jie Ma

**Affiliations:** 1 Department of Pediatric Neurosurgery, Shanghai Xin Hua Hospital Affiliated To Shanghai Jiaotong University, School of Medicine, No. 1665 Kongjiang Road, Shanghai, 200092 China; 2 State Key Laboratory of Oncogenes and Related Genes, Shanghai Cancer Institute, Renji Hospital, Shanghai Jiaotong University School of Medicine, No. 25/Ln 2200 Xie Tu Road, Shanghai, 200032 China; 3 Key Laboratory of Systems Biomedicine (Ministry of Education), Shanghai Center for Systems Biomedicine, Shanghai Jiao Tong University, Shanghai, 200240 China

## Correction to: J Nanobiotechnol 18:169 (2020) https://doi.org/10.1186/s12951-020-00729-9

Following publication of the original article [1], the authors identified an inadvertent error. Figure 2f and e were partially duplicated, an error which was possibly made during image compilation. It is therefore necessary to correct Fig. 2f. The corrected Figure 2f and figure caption are given below. The correction of this figure does not affect the results and conclusion. All authors agree to this correction and apologize for this error. The original full field view of Figure 2f is also provided in supplementary information Fig S1.

The authors alsofound that the cells in the left three columns (the last row of Fig. 3) were inconsistent with those in the right three columns. In order to facilitate the readers’ understanding of that part of the content and avoid any possible confusion, we hereby provide the cells consistent with the right three columns as a supplement. The supplementary pictures are shown below in Fig. 3 (the last row). The original full field view of the last row of Fig. 3 is also provided in supplementary information Fig S2.

In summary, we provide a correction of Figure 2f and a supplementary image for the last row of Fig. 3 as follows:

1. The correction of Fig. 2f:

**Fig. 2:**
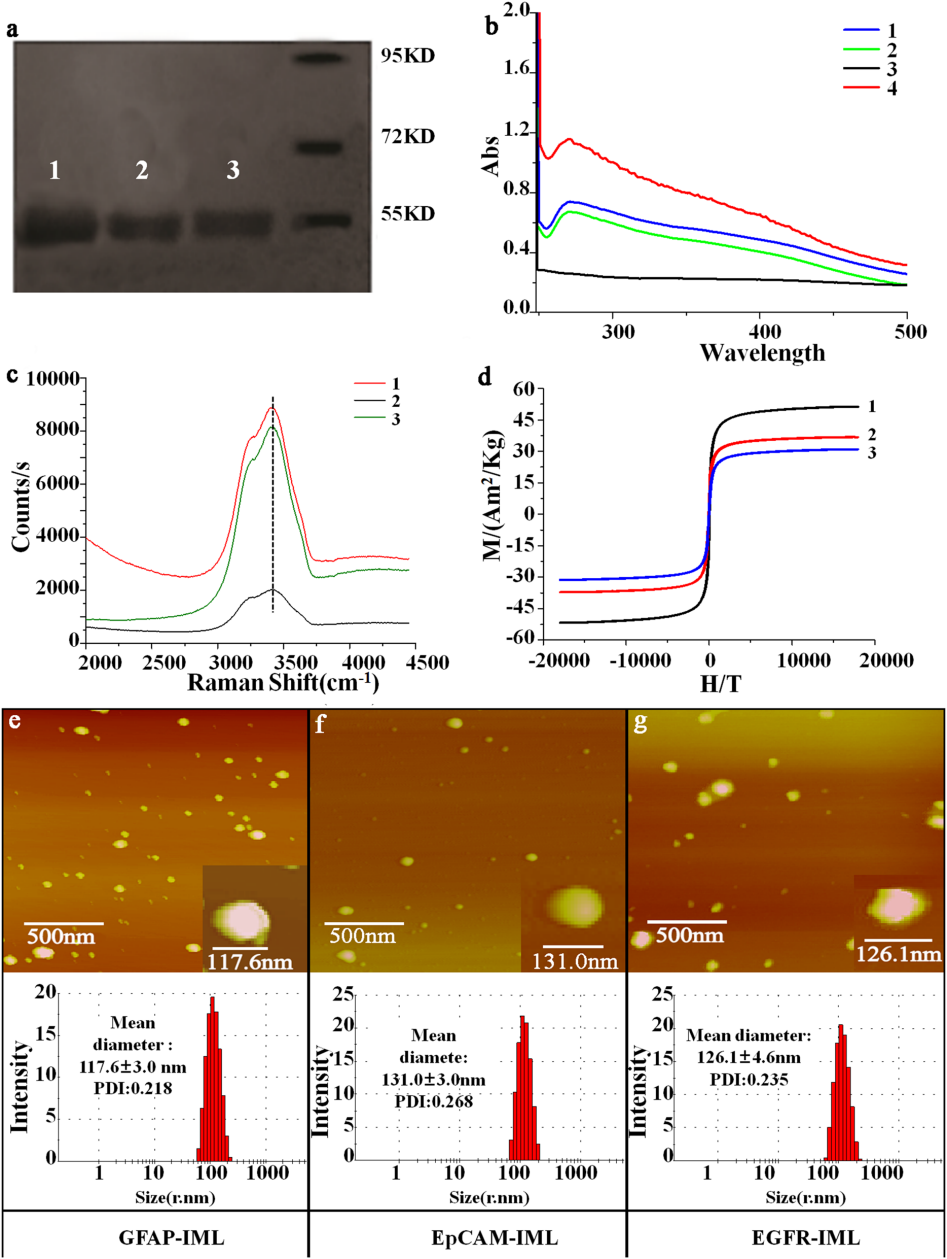
Material characteristics of the three IMLs. **a** Protein electrophoresis. Lanes 1–3 were GFAP, GFAP-GHDC, and GFAP-IMLs, respectively; **b** UV spectra. Lines 1–4 indicate the GFAP, GFAP-GHDC, Fe_3_O_4_ raw magnetic beads and GFAP-IMLs; **c** Raman spectra. Lines 1–3 indicate the GFAP, GFAP-GHDC, and GFAP-IMLs; **d** VSM magnetization curves. Lines 1–3 demonstrate the Fe3O4 raw magnetic beads, magnetic liposomes, and antibody-IMLs, respectively. **e** Upper: AFM topographic image and below: particle size distribution of GFAP-IMLs; **f** Upper: AFM topographic image and below: particle size distribution of EpCAM-IMLs; **g** Upper: AFM topographic image and below: particle size distribution of EGFR-IMLs

Supplementary information Fig S1:
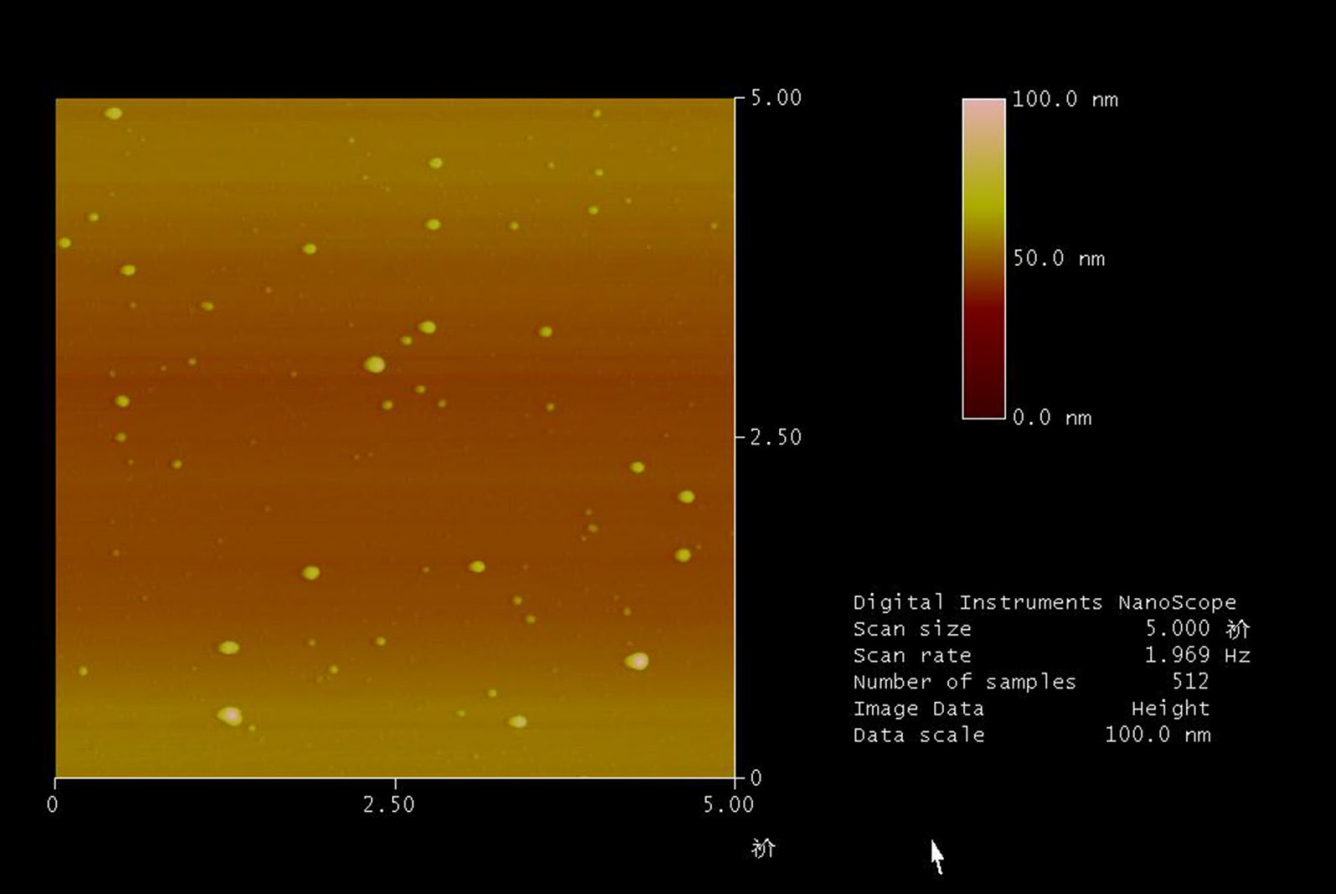


2. The supplementary image for Fig. 3 (the last row):
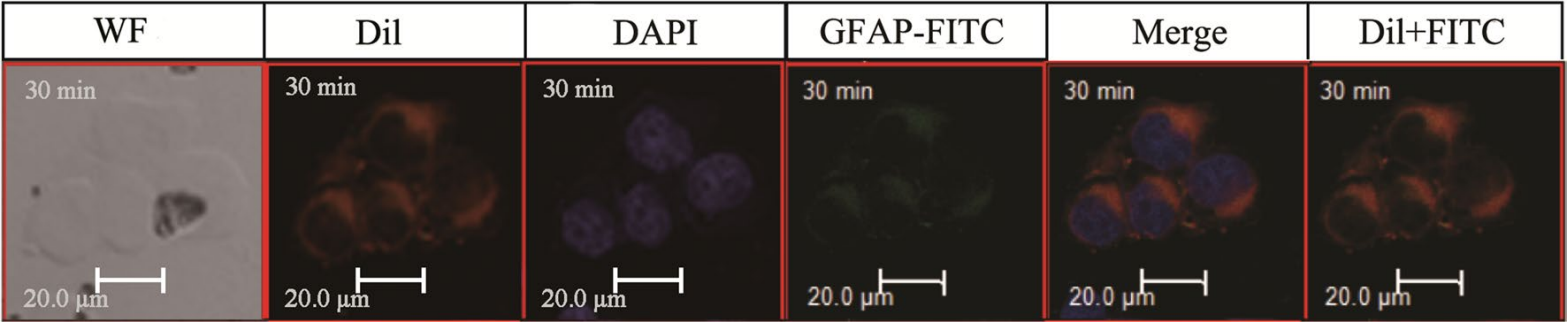


Supplementary information Fig S2:
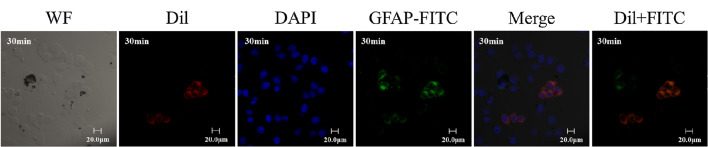


The authors apologize for these errors and any inconvenience to the reader.
