# Supplementary material for: Cytoplasm protein GFAP magnetic beads construction and application as cell separation target for brain tumors
Source: J Nanobiotechnology. 2020 Nov 18;18:169. doi: 10.1186/s12951-020-00729-9 (PMC7673097; doi:10.1186/s12951-020-00729-9)
Supplement: Supplementary file 1 — Additional file 1. Additional figures and tables, [file 12951_2020_729_MOESM1_ESM.doc]

# Cytoplasm Protein GFAP Magnetic Beads Construction and Application as Cell Separation target for Brain Tumors

Yang Zhao,^#a^ Feng Jiang,^#a^ Qinhua Wang,^#a^ Baocheng Wang,^a^ Yipeng Han,^a^ Jian Yang,^a^ Jiajia Wang,^a^ Kai Wang,^b^ Junping Ao,^b^ Xunxiang Guo,^c^ Xiaofei Liang^*b,c^ and Jie Ma^*a^

*^a^ Department of Pediatric Neurosurgery, Shanghai Xin Hua Hospital Affiliated to Shanghai Jiaotong University, School of Medicine, No. 1665 Kongjiang Road, Shanghai 200092, China. E-mail: majie@xinhuamed.com.cn*

*^b^ State Key Laboratory of Oncogenes and Related Genes, Shanghai Cancer Institute, Renji Hospital, Shanghai Jiaotong University School of Medicine, No. 25/Ln 2200 Xie Tu Road, Shanghai 200032, China. E-mail:* [*xfliang@shsci.org*](mailto:xfliang@shsci.org)

*^c^ Key Laboratory of Systems Biomedicine (Ministry of Education), Shanghai Center for Systems Biomedicine, Shanghai Jiao Tong University, Shanghai 200240, China*

*^*^* *Authors to whom correspondence should be addressed*

*^#^ Yang Zhao, Feng Jiang and Qinhua Wang contributed equally to this work*

**Supplementary explanation of IMLs preparation process**

The flow chart of CTC isolation in PB and CSF from children with brain tumor by IMLs was listed in Figure 1. PB was collected and centrifuged at 1000g for 10mins. The upper - middle layer and the lower layer of CSF were removed and IML was added, respectively. Magnetic separation was performed after binding process. The isolated CTCs were stained by immunofluorescence dye and subjected to fluorescence microscope. The results were statistically analyzed and a diagnostic report was prepared accordingly.

**Supplementary Figures**


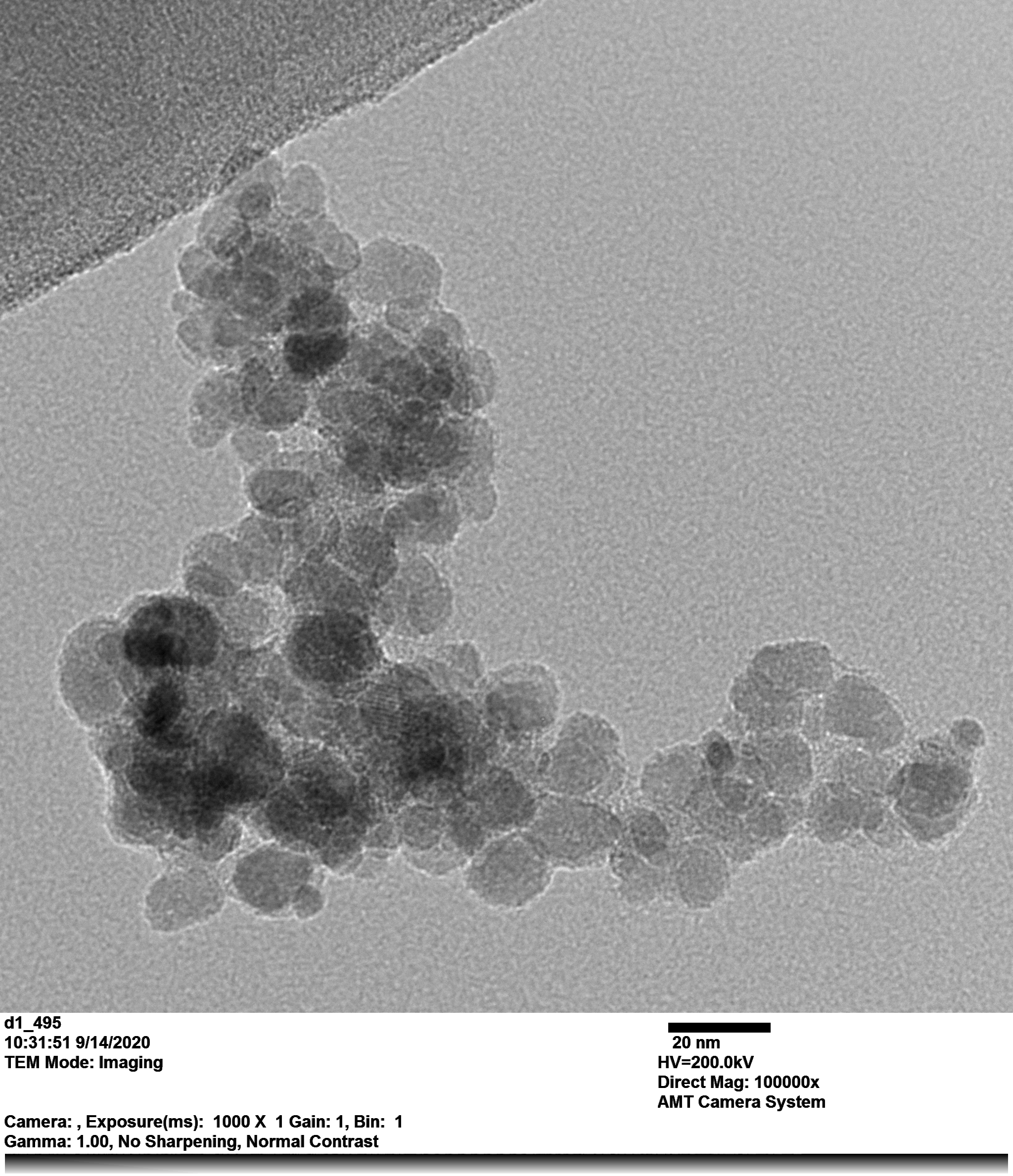


**Figure. S1** TEM image of GFAP-IMLs.

**
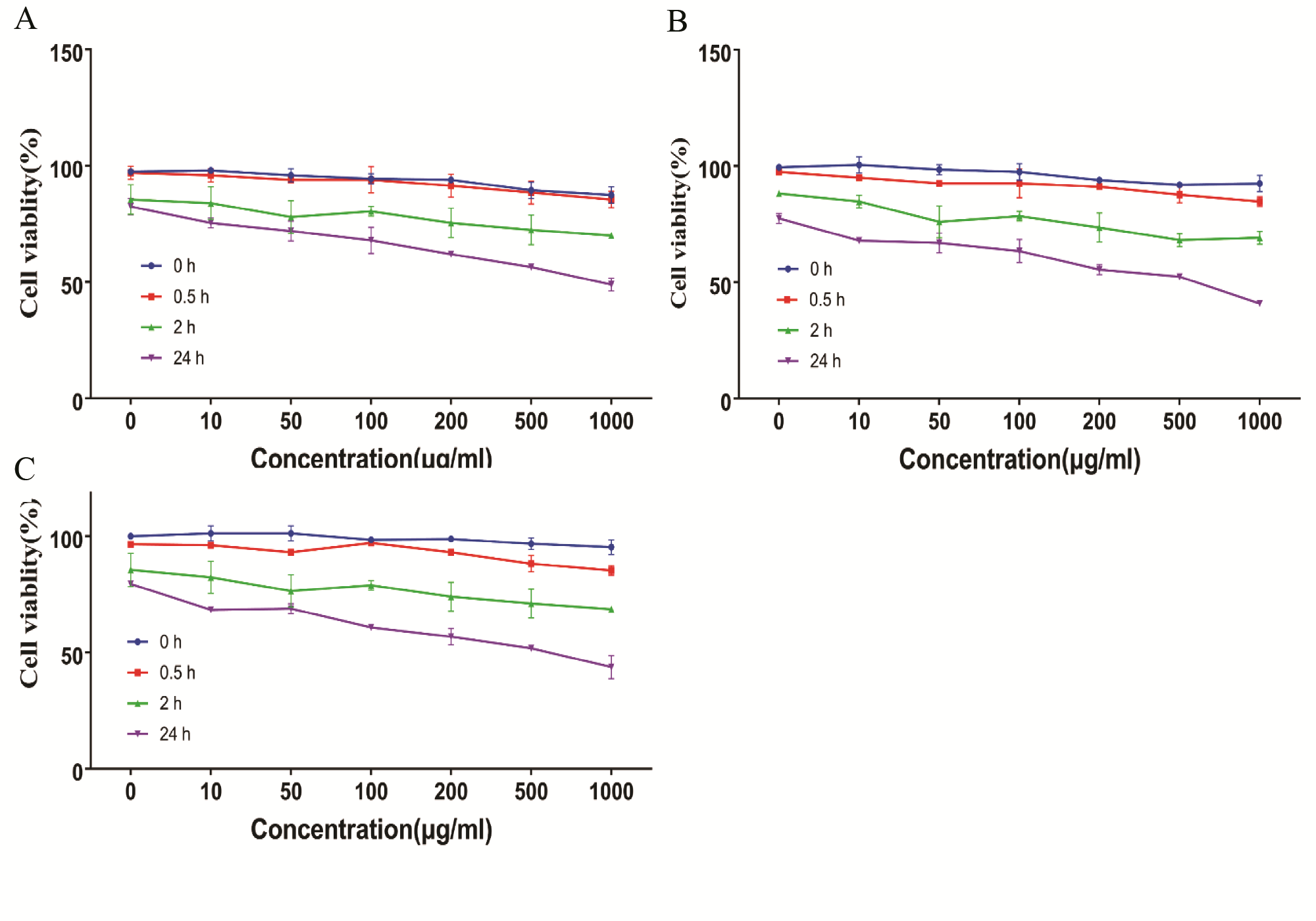
**

**Figure. S2** A: Cell viability curve of U251 cells treated by GFAP-IMLs. X-axis represents concentration of GFAP-IMLs(μg/ml), Y-axis represents cell viability (%). Curves with four colors refer to various treating time points; B: Cell viability curve of U251 cells treated by EpCAM-IML. X-axis represents concentration of EpCAM-IML(μg/ml), Y-axis represents cell viability (%). Curves with four colors refer to various treating time points; C: Cell viability curve of U251 cells treated by EGFR-IML. X-axis represents concentration of EGFR-IML(μg/ml), Y-axis represents cell viability (%). Curves with four colors refers various treating time points.


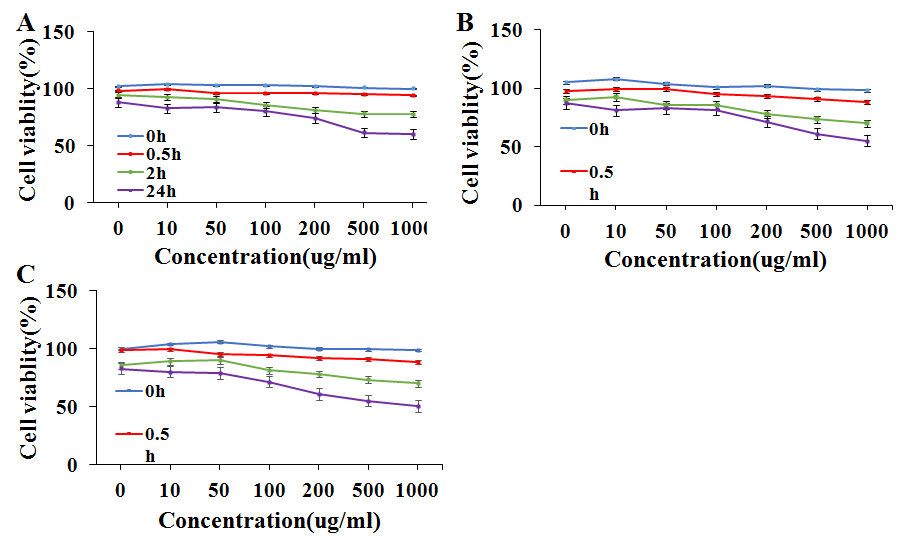


**Figure. S3** A: Cell viability curve of D425 cells treated by GFAP-IMLs. X-axis represents concentration of GFAP-IMLs(μg/ml), Y-axis represents cell viability (%). Curves with four colors refer to various treating time points; B: Cell viability curve of D425 cells treated by EpCAM-IML. X-axis represents concentration of EpCAM-IML(μg/ml), Y-axis represents cell viability (%). Curves with four colors refer to various treating time points; C: Cell viability curve of D425 cells treated by EGFR-IML. X-axis represents concentration of EGFR-IML(μg/ml), Y-axis represents cell viability (%). Curves with four colors refers various treating time points.


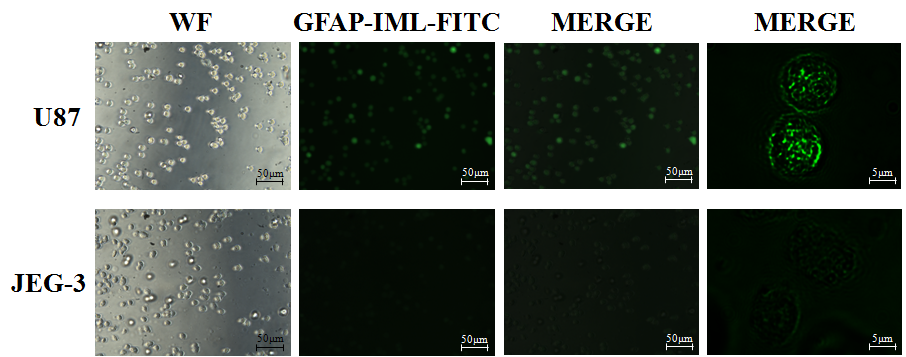


**Figure. S4** FITC-GFAP-IMLs interacted with U87 and JEG-3 cells. The FITC-GFAP-IMLs uptake was compared between U87 cells with high GFAP expression and JEG-3 cells without GFAP expression. The interaction time of GFAP-IMLs with cells was 25min.

**
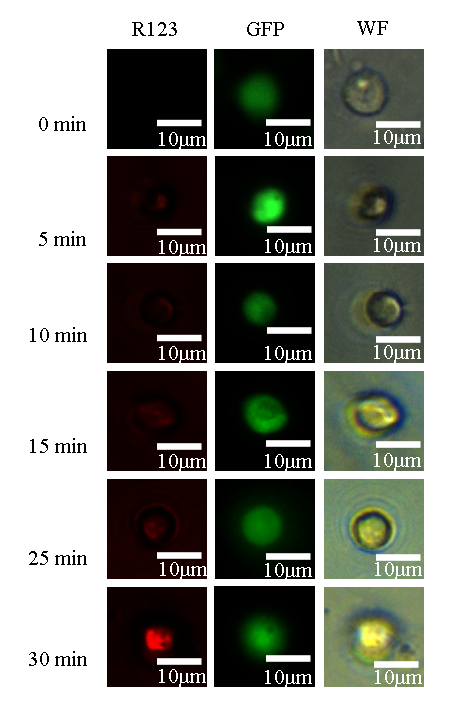
**

**Figure. S5** GFAP-IMLs interacted with U87 cells.

R123 refers to rhodamine 123, GFP refers to the spontaneous fluorescence of GFP protein in U87 cells, and WF refers to the white field. 0min，5min，10min，15min，25min and 30min refered to the incubation time of GFAP-IMLs-rhodamine 123 and U87 cells.


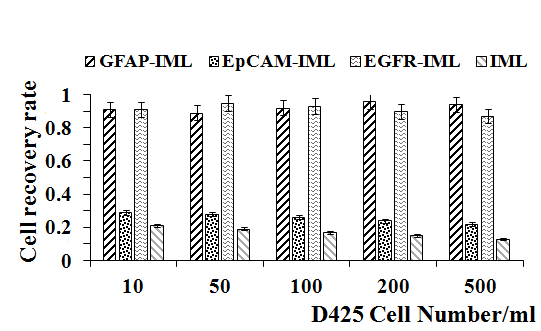


**Figure. S6** Comparison of the cell capture efficiency among the three IMLs in PBS.


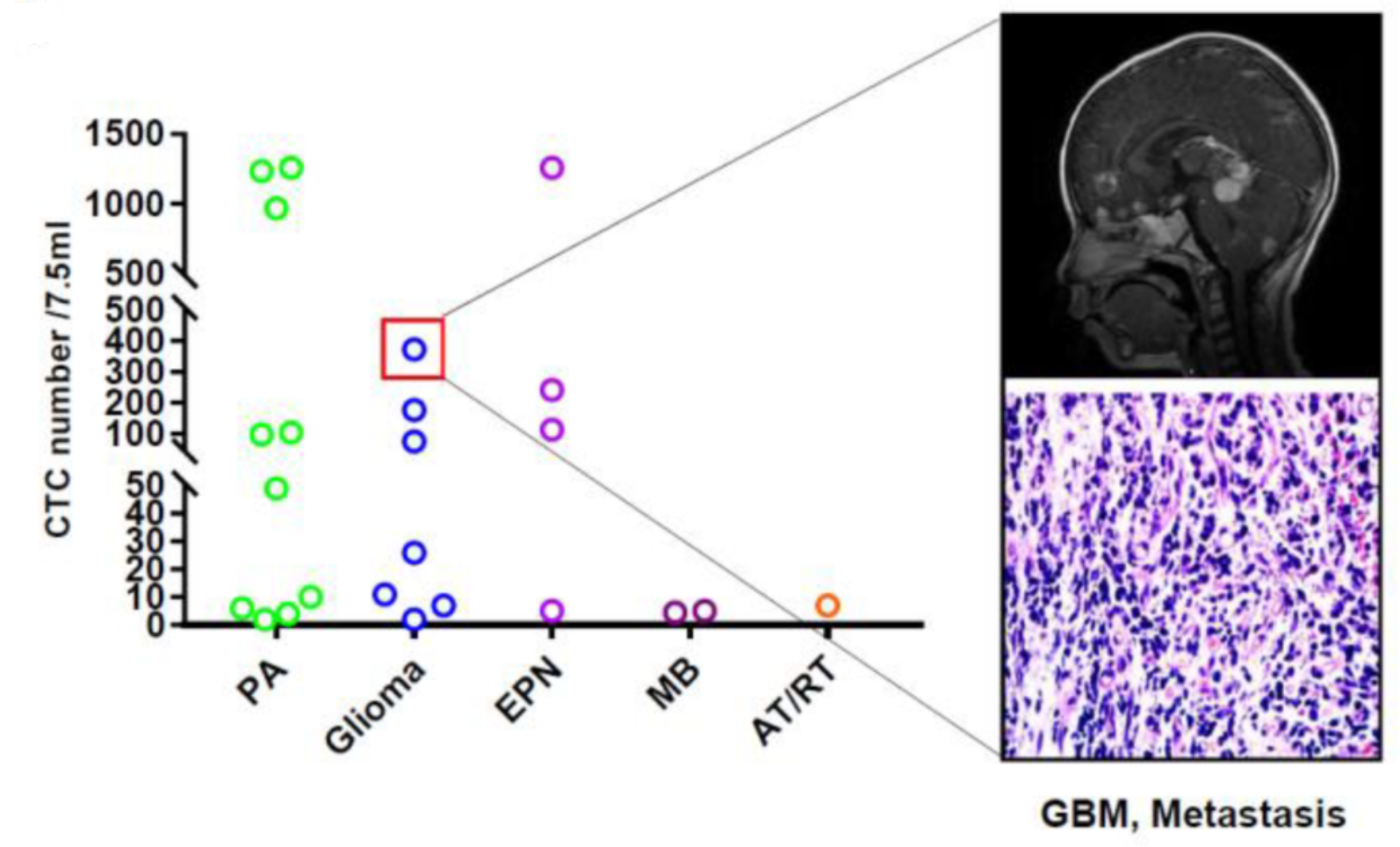


**Figure. S7.** Dot-plot displays data distribution of captured CTC. The point indicated by red frame refers one case with cerebellar glioblastoma with CSF dissemination as presented by sagittal MRI T1-contrast signal (Upper) as well as representative H&E staining (400x).


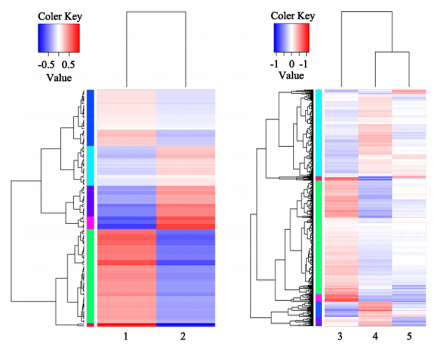


**Figure. S8** Clustering of differentially expressed genes of the 5 samples was carried out by log10 (FPKM+1) value, with red representing high-expressed genes and blue representing low-expressed genes. The color ranges from blue to red, indicating higher gene expression.

**Supplementary Table 1.**

**Detailed information on clinical characters of each patient recruited in this study.**

| Sample | Gender | Age (year) | Sample Type | Sample Volume | Magnetic Bead sorted cell  EpCAM EGFR GFAP | | | Total CTC | Pathological Diagnosis | WHO Grade | Location |
| --- | --- | --- | --- | --- | --- | --- | --- | --- | --- | --- | --- |
| **1** | M | 2 | Blood | 7.5ml | 1 | 0 | 0 | 1 | Glioblastoma Multiforme(GBM) | IV | Right Tempro-Paarietal Lobe (RTP) |
|  |  |  | CSF | 7.5ml | 8 | 13 | 5 | 26 |  |  |  |
| **2** | M | *8* | Blood | 7.5ml | 1 | 1 | 2 | 4 | Anaplastic Ependymoma (AEPN) | III | Right Tempro-Parietal-Occipital Lobe (RTPO) |
|  |  |  | CSF | 7.5ml | 99 | 71 | 70 | 241 |  |  |  |
| **3** | M | 2 | Blood | 7.5ml | 1 | 0 | 0 | 1 | Anaplastic Ependymoma (AEPN) | III | Fourth Ventricle (FV) |
|  |  |  | CSF | 7.5ml | 169 | 107 | 311 | 587 |  |  |  |
| **4** | F | 6 | Blood | 7.5ml | 1 | 2 | 3 | 6 | Pilocytic Astrocytoma (PA) | I | Supra-Sellar Cistern |
|  |  |  | CSF | 7.5ml | 7 | 5 | 5 | 17 |  |  |  |
| **5** | M | 1 month | Blood | 7.5ml | 1 | 0 | 0 | 1 | Anaplastic Astrocytoma | I | Left Ventrical (LV) |
|  |  |  | CSF | 7.5ml | 31 | 28 | 127 | 186 |  |  |  |
| **6** | M | 2 | Blood | 7.5ml | 1 | 0 | 0 | 1 | Pilocytic Astrocytoma (PA) | I | Fourth Ventricle (FV) |
|  |  |  | CSF | 7.5ml | 11 | 0 | 1 | 2 |  |  |  |
| **7** | F | 3 | Blood | 7.5ml | 1 | 0 | 0 | 1 | Medulloblastoma (MB) | IV | Fourth Ventricle (FV) |
|  |  |  | CSF | 7.5ml | 5 | 3 | 4 | 12 |  |  |  |
| **8** | M | 3 | Blood | 7.5ml | 3 | 2 | 2 | 7 | Pilocytic Astrocytoma (PA) | I | Right Basal Ganglia (RBG) |
|  |  |  | CSF | 7.5ml | 30 | 52 | 94 | 176 |  |  |  |
| **9** | M | 2 | Blood | 7.5ml | 2 | 1 | 2 | 5 | Pilocytic Astrocytoma (PA) | I | Right Tempro-Parietal-Occipital Lobe (RTPO) |
|  |  |  | CSF | 7.5ml | 46 | 39 | 44 | 129 |  |  |  |
| **10** | M | 5 | Blood | 7.5ml | 3 | 1 | 0 | 4 | Pilocytic Astrocytoma (PA) | I | Fourth Ventricle (FV) |
|  |  |  | CSF | 7.5ml | 0 | 3 | 0 | 3 |  |  |  |
| **11** | F | 1 | Blood | 7.5ml | 1 | 1 | 0 | 2 | Pilocytic Astrocytoma (PA) | I | Fourth Ventricle (FV) |
|  |  |  | CSF | 7.5ml | 0 | 2 | 3 | 5 |  |  |  |
| **12** | M | 8 month | Blood | 7.5ml | 0 | 1 | 1 | 2 | AT0RT | IV | Left Ventrical (LV) |
|  |  |  | CSF | 7.5ml | 3 | 1 | 3 | 7 |  |  |  |
| **13** | F | 7 | Blood | 7.5ml | 0 | 0 | 0 | 0 | Ependymoma (EPN) | II | Third Ventricle (TV) |
|  |  |  | CSF | 7.5ml | 0 | 0 | 5 | 5 |  |  |  |
| **14** | F | 6 | Blood | 7.5ml | 2 | 0 | 0 | 2 | DIPG,H3K27M (+) | IV | Brain Stem |
|  |  |  | CSF | 7.5ml | 0 | 0 | 0 | 2 |  |  |  |
| **15** | F | 4 | Blood | 7.5ml | 2 | 3 | 0 | 5 | Pilocytic Astrocytoma (PA) | I | Sellar |
|  |  |  | CSF | 7.5ml | 17 | 12 | 20 | 49 |  |  |  |
| **16** | F | 9 | Blood | 7.5ml | 1 | 1 | 0 | 2 | Pilocytic Astrocytoma (PA) | I | Cerebellar |
|  |  |  | CSF | 7.5ml | 19 | 39 | 42 | 100 |  |  |  |
| **17** | F | 6 | Blood | 7.5ml | 0 | 0 | 1 | 1 | DIPG,H3K27M (+) | IV | Brain Stem |
|  |  |  | CSF | 7.5ml | 0 | 0 | 4 | 4 |  |  |  |
| **18** | F | 5 | Blood | 7.5ml | 4 | 3 | 6 | 13 | Glioblastoma Multiforme(GBM) | IV | Brain Stem |
|  |  |  | CSF | 7.5ml | 24 | 18 | 55 | 97 |  |  |  |
| **19** | F | 8 month | Blood | 7.5ml | 3 | 5 | 8 | 16 | Medulloblastoma (MB) | IV | Fourth Ventricle (FV) |
|  |  |  | CSF | 7.5ml | 33 | 15 | 59 | 107 |  |  |  |
| **20** | M | 4 | Blood | 7.5ml | 2 | 2 | 5 | 9 | Pilocytic Astrocytoma (PA) | I | T9-11 |
|  |  |  | CSF | 7.5ml | 20 | 16 | 38 | 74 |  |  |  |
| **21** | M | 2 | Blood | 7.5ml | 3 | 2 | 0 | 5 | Anaplastic Ependymoma (AEPN) | III | Fourth Ventricle (FV) |
|  |  |  | CSF | 7.5ml | 2 | 3 | 108 | 113 |  |  |  |
| **22** | M | 3 | Blood | 7.5ml | 3 | 2 | 2 | 7 | Glioblastoma Multiforme(GBM) | IV | Cerebellar,Metastasis |
|  |  |  | CSF | 7.5ml | 30 | 52 | 94 | 176 |  |  |  |
| **23** | M | 4 | Blood | 7.5ml | 4 | 5 | 4 | 13 | DIPG,H3K27M C+) | IV | Brain Stem |
|  |  |  | CSF | 7.5ml | 3 | 6 | 66 | 75 |  |  |  |
| **24** | M | 7 | Blood | 7.5ml | 2 | 1 | 3 | 6 | Pilocytic Astrocytoma (PA) | I | Cerebellar |
|  |  |  | CSF | 7.5ml | 30 | 24 | 50 | 104 |  |  |  |
| **25** | M | 7 | Blood | 7.5ml | 1 | 0 | 2 | 3 | DIPG,H3K27M C+) | IV | Brain Stem |
|  |  |  | CSF | 7.5ml | 1 | 2 | 2 | 5 |  |  |  |
| **26** | F | 2 | Blood | 7.5ml | 1 | 2 | 2 | 5 | Medulloblastoma (MB) | III | Cerebellar |
|  |  |  | CSF | 7.5ml | 2 | 3 | 5 | 10 |  |  |  |
| **27** | M | 6 | Blood | 7.5ml | 80 | 117 | 245 | 442 | Medulloblastoma (MB) | IV | Cerebellar |
|  |  |  | CSF | 7.5ml | 41 | 62 | 86 | 189 |  |  |  |
| **28** | F | 13 | Blood | 7.5ml | 5 | 9 | 10 | 24 | Chiari Malformation | / | / |
|  |  |  | CSF | 7.5ml | 5 | 12 | 18 | 35 |  |  |  |
| **29** | F | 5 | Blood | 7.5ml | 3 | 2 | 4 | 9 | DIPG,H3K27M C+) | IV | Brain Stem |
|  |  |  | CSF | 7.5ml | 3 | 2 | 8 | 13 |  |  |  |
| **30** | M | 2 | Blood | 7.5ml | 120 | 350 | 400 | 870 | Craniostenosis |  | Control |
|  |  |  | CSF | 7.5ml | 0 | 0 | 0 | 0 |  |  |  |
| **31** | F | 1 | Blood | 7.5ml | 0 | 1 | 1 | 01 | Scalp Mass |  |  |
|  |  |  | CSF | 7.5ml | 2 | 1 | 5 | 8 |  |  |  |
| **32** | F | 1 | Blood | 7.5ml | 1 | 0 | 1 | 2 | Craniostenosis |  |  |
|  |  |  | CSF | 7.5ml | 2 | 0 | 0 | 2 |  |  |  |

**Supplementary Table 2**. Clinical statistics of brain tumor patients and the number of isolated CTCs.

| **Sample** | **Gender** | **Age (year)** | **Sample Type** | **Sample Volume** | **Magnetic Bead sorted cell** | | **Total CTC** | **Pathological Diagnosis** | **WHO Grade** |
| --- | --- | --- | --- | --- | --- | --- | --- | --- | --- |
|  |  |  |  |  | **EGFR** | **GFAP** |  |  |  |
| 1 | F | 4 | Blood | 7.5ml | 1 | 0 | 1 | Anaplastic Ependymoma (AEPN) | IV |
|  |  |  | CSF | 7.5ml | 120 | 0 | 120 |  |  |
| 2 | F | 2 | Blood | 7.5ml | 0 | 0 | 0 | Glioblastoma Multiforme(GBM) | IV |
|  |  |  | CSF | 7.5ml | 13 | 5 | 18 |  |  |
| 3 | F | 9 | Blood | 7.5ml | 1 | 2 | 3 | Anaplastic Ependymoma (AEPN) | III |
|  |  |  | CSF | 7.5ml | 71 | 70 | 141 |  |  |
| 4 | F | 4 | Blood | 7.5ml | 6 | 6 | 12 | DIPG,H3K27M (+) | IV |
|  |  |  | CSF | 7.5ml | 8 | 76 | 84 |  |  |
| 5 | M | 7 | Blood | 7.5ml | 0 | 0 | 0 | Pilocytic Astrocytoma (PA) | I |
|  |  |  | CSF | 7.5ml | 52 | 38 | 90 |  |  |
| 6 | F | 1month | Blood | 7.5ml | 0 | 0 | 0 | Pilocytic Astrocytoma (PA) | II |
|  |  |  | CSF | 7.5ml | 35 | 85 | 120 |  |  |
| 7 | M | 3 | Blood | 7.5ml | 0 | 0 | 0 | Medulloblastoma (MB) | IV |
|  |  |  | CSF | 7.5ml | 2 | 3 | 5 |  |  |
| 8 | F | 2 | Blood | 7.5ml | 0 | 0 | 0 | Anaplastic Ependymoma (AEPN) | III |
|  |  |  | CSF | 7.5ml | 21 | 62 | 83 |  |  |
| 9 | F | 3 | Blood | 7.5ml | 0 | 0 | 0 | Pilocytic Astrocytoma (PA) | I |
|  |  |  | CSF | 7.5ml | 0 | 1 | 1 |  |  |
| 10 | M | 4 | Blood | 7.5ml | 2 | 2 | 3 | Skull fracture | / |
|  |  |  | CSF | 7.5ml | 14 | 10 | 24 |  |  |
| 11 | M | 10 | Blood | 7.5ml | 0 | 4 | 4 | Germ-cell tumor | IV |
|  |  |  | CSF | 7.5ml | 46 | 86 | 132 |  |  |
| 12 | M | 1 | Blood | 7.5ml | 4 | 3 | 7 | Scalp Mass | / |
|  |  |  | CSF | 7.5ml | 18 | 14 | 32 |  |  |
| 13 | F | 2 | Blood | 7.5ml | 3 | 2 | 5 | Scalp Mass | / |
|  |  |  | CSF | 7.5ml | 9 | 5 | 14 |  |  |
| 14 | F | 5 | Blood | 7.5ml | 2 | 0 | 2 | Osteofibrous dysplasia | / |
|  |  |  | CSF | 7.5ml | 5 | 6 | 11 |  |  |
| 15 | F | 2 | Blood | 7.5ml | 1 | 0 | 1 | Craniostenosis | / |
|  |  |  | CSF | 7.5ml | 6 | 2 | 8 |  |  |
| 16 | F | 3 | Blood | 7.5ml | 0 | 0 | 0 | Pilocytic Astrocytoma (PA) | I |
|  |  |  | CSF | 7.5ml | 24 | 33 | 67 |  |  |
| 17 | F | 3 | Blood | 7.5ml | 2 | 4 | 6 | Pilocytic Astrocytoma (PA) | I |
|  |  |  | CSF | 7.5ml | 49 | 26 | 75 |  |  |
| 18 | F | 6 | Blood | 7.5ml | 2 | 0 | 2 | Pilocytic Astrocytoma (PA) | I |
|  |  |  | CSF | 7.5ml | 58 | 117 | 175 |  |  |
| 19 | M | 1 | Blood | 7.5ml | 2 | 0 | 2 | Pilocytic Astrocytoma (PA) | I |
|  |  |  | CSF | 7.5ml | 4 | 6 | 10 |  |  |
| 20 | F | 1 | Blood | 7.5ml | 2 | 2 | 4 | Atypical teratoma | IV |
|  |  |  | CSF | 7.5ml | 1 | 3 | 4 |  |  |
| 21 | M | 7 | Blood | 7.5ml | 0 | 0 | 0 | Anaplastic Ependymoma (AEPN) | II |
|  |  |  | CSF | 7.5ml | 0 | 5 | 5 |  |  |
| 22 | M | 6 | Blood | 7.5ml | 0 | 1 | 1 | DIPG,H3K27M C+) | IV |
|  |  |  | CSF | 7.5ml | 0 | 4 | 4 |  |  |
| 23 | M | 4month | Blood | 7.5ml | 2 | 3 | 5 | Pilocytic Astrocytoma (PA) | I |
|  |  |  | CSF | 7.5ml | 17 | 20 | 37 |  |  |
| 24 | M | 6 | Blood | 7.5ml | 2 | 0 | 2 | DIPG,H3K27M C+) | IV |
|  |  |  | CSF | 7.5ml | 0 | 2 | 2 |  |  |
| 25 | M | 10 | Blood | 7.5ml | 1 | 1 | 2 | Pilocytic Astrocytoma (PA) | I |
|  |  |  | CSF | 7.5ml | 39 | 42 | 81 |  |  |
| 26 | M | 5 | Blood | 7.5ml | 5 | 8 | 13 | Glioblastoma Multiforme(GBM) | IV |
|  |  |  | CSF | 7.5ml | 41 | 56 | 97 |  |  |
| 27 | M | 1 | Blood | 7.5ml | 6 | 10 | 16 | Medulloblastoma (MB) | IV |
|  |  |  | CSF | 7.5ml | 59 | 48 | 107 |  |  |
| 28 | F | 4 | Blood | 7.5ml | 4 | 5 | 9 | Pilocytic Astrocytoma (PA) | I |
|  |  |  | CSF | 7.5ml | 36 | 38 | 74 |  |  |
| 29 | F | 2 | Blood | 7.5ml | 3 | 2 | 5 | Anaplastic Ependymoma (AEPN) | III |
|  |  |  | CSF | 7.5ml | 84 | 43 | 127 |  |  |
| 30 | F | 3 | Blood | 7.5ml | 3 | 4 | 7 | Glioblastoma Multiforme(GBM) | IV |
|  |  |  | CSF | 7.5ml | 62 | 74 | 136 |  |  |
| 31 | F | 2 | Blood | 7.5ml | 1 | 2 | 3 | Melanoma | IV |
|  |  |  | CSF | 7.5ml | 39 | 44 | 83 |  |  |
| 32 | F | 5 | Blood | 7.5ml | 4 | 9 | 13 | DIPG,H3K27M C+) | IV |
|  |  |  | CSF | 7.5ml | 9 | 66 | 75 |  |  |
| 33 | M | 1 | Blood | 7.5ml | 1 | 1 | 2 | Craniostenosis | / |
|  |  |  | CSF | 7.5ml | 2 | 0 | 2 |  |  |
| 34 | F | 6 | Blood | 7.5ml | 3 | 3 | 6 | Pilocytic Astrocytoma (PA) | I |
|  |  |  | CSF | 7.5ml | 24 | 50 | 74 |  |  |
| 35 | M | 3 | Blood | 7.5ml | 0 | 1 | 1 | Scalp Mass | / |
|  |  |  | CSF | 7.5ml | 3 | 5 | 8 |  |  |
| 36 | M | 7 | Blood | 7.5ml | 3 | 3 | 6 | Pilocytic Astrocytoma (PA) | I |
|  |  |  | CSF | 7.5ml | 12 | 5 | 17 |  |  |
| 37 | M | 4 | Blood | 7.5ml | 0 | 0 | 0 | Medulloblastoma (MB) | IV |
|  |  |  | CSF | 7.5ml | 8 | 4 | 12 |  |  |
| 38 | F | 7 | Blood | 7.5ml | 1 | 2 | 3 | DIPG,H3K27M C+) | IV |
|  |  |  | CSF | 7.5ml | 5 | 0 | 5 |  |  |
| 39 | M | 4 | Blood | 7.5ml | 1 | 3 | 4 | Pilocytic Astrocytoma (PA) | I |
|  |  |  | CSF | 7.5ml | 2 | 4 | 6 |  |  |
| 40 | M | 6 | Blood | 7.5ml | 1 | 0 | 1 | DIPG,H3K27M C+) | IV |
|  |  |  | CSF | 7.5ml | 3 | 0 | 3 |  |  |
| 41 | F | 11 | Blood | 7.5ml | 4 | 3 | 7 | Encephalitis | / |
|  |  |  | CSF | 7.5ml | 2 | 3 | 5 |  |  |
| 42 | F | 2 | Blood | 7.5ml | 0 | 0 | 0 | Medulloblastoma (MB) | IV |
|  |  |  | CSF | 7.5ml | 2 | 4 | 6 |  |  |
| 43 | M | 5 | Blood | 7.5ml | 0 | 0 | 0 | Medulloblastoma (MB) | IV |
|  |  |  | CSF | 7.5ml | 4 | 0 | 4 |  |  |
| 44 | M | 6 | Blood | 7.5ml | 5 | 4 | 9 | DIPG,H3K27M C+) | IV |
|  |  |  | CSF | 7.5ml | 5 | 8 | 13 |  |  |
| 45 | M | 2 | Blood | 7.5ml | 2 | 3 | 5 | Medulloblastoma (MB) | IV |
|  |  |  | CSF | 7.5ml | 3 | 5 | 8 |  |  |
| 46 | F | 2 | Blood | 7.5ml | 2 | 3 | 5 | Craniostenosis | / |
|  |  |  | CSF | 7.5ml | 0 | 0 | 0 |  |  |
| 47 | F | 6 | Blood | 7.5ml | 4 | 7 | 11 | Medulloblastoma (MB) | IV |
|  |  |  | CSF | 7.5ml | 62 | 86 | 148 |  |  |
| 48 | M | 14 | Blood | 7.5ml | 9 | 10 | 19 | Chiari Malformation | / |
|  |  |  | CSF | 7.5ml | 12 | 18 | 30 |  |  |
| 49 | F | 6 | Blood | 7.5ml | 1 | 3 | 4 | DIPG,H3K27M C+) | IV |
|  |  |  | CSF | 7.5ml | 0 | 0 | 0 |  |  |
| 50 | M | 6 | Blood | 7.5ml | 0 | 0 | 0 | DIPG,H3K27M C+) | IV |
|  |  |  | CSF | 7.5ml | 9 | 5 | 14 |  |  |
| 51 | M | 3 | Blood | 7.5ml | 5 | 3 | 8 | Anaplastic Ependymoma (AEPN) | III |
|  |  |  | CSF | 7.5ml | 16 | 4 | 20 |  |  |
| 52 | M | 7 | Blood | 7.5ml | 1 | 2 | 3 | DIPG,H3K27M C+) | IV |
|  |  |  | CSF | 7.5ml | 13 | 11 | 24 |  |  |
| 53 | F | 10 | Blood | 7.5ml | 2 | 1 | 3 | Medulloblastoma (MB) | IV |
|  |  |  | CSF | 7.5ml | 4 | 2 | 6 |  |  |
| 54 | F | 5 | Blood | 7.5ml | 2 | 4 | 6 | Pilocytic Astrocytoma (PA) | II |
|  |  |  | CSF | 7.5ml | 0 | 0 | 0 |  |  |
| 55 | F | 8 | Blood | 7.5ml | 2 | 1 | 3 | Pilocytic Astrocytoma (PA) | I |
|  |  |  | CSF | 7.5ml | 2 | 3 | 5 |  |  |
| 56 | M | 4 | Blood | 7.5ml | 1 | 3 | 4 | Pilocytic Astrocytoma (PA) | I |
|  |  |  | CSF | 7.5ml | 2 | 4 | 6 |  |  |
| 57 | F | 1 | Blood | 7.5ml | 3 | 12 | 15 | Encephalitis | / |
|  |  |  | CSF | 7.5ml | 5 | 5 | 10 |  |  |
| 58 | F | 5 | Blood | 7.5ml | 6 | 2 | 8 | Medulloblastoma (MB) | IV |
|  |  |  | CSF | 7.5ml | 2 | 2 | 4 |  |  |
| 59 | F | 4 | Blood | 7.5ml | 5 | 2 | 7 | Anaplastic Ependymoma (AEPN) | III |
|  |  |  | CSF | 7.5ml | 4 | 2 | 6 |  |  |
| 60 | F | 11 | Blood | 7.5ml | 3 | 3 | 6 | Germ-cell tumor | IV |
|  |  |  | CSF | 7.5ml | 2 | 2 | 4 |  |  |
| 61 | F | 13 | Blood | 7.5ml | 4 | 9 | 13 | Collision tumor | I |
|  |  |  | CSF | 7.5ml | 7 | 3 | 10 |  |  |
| 62 | F | 1 | Blood | 7.5ml | 12 | 6 | 18 | Choroid plexus papilloma | I |
|  |  |  | CSF | 7.5ml | 2 | 4 | 6 |  |  |
| 63 | F | 3 | Blood | 7.5ml | 8 | 4 | 12 | Low grade glioma (LGG) | II |
|  |  |  | CSF | 7.5ml | 6 | 1 | 7 |  |  |
| 64 | F | 8 | Blood | 7.5ml | 7 | 14 | 11 | Embryonal tumor | IV |
|  |  |  | CSF | 7.5ml | 0 | 0 | 0 |  |  |
| 65 | M | 9 | Blood | 7.5ml | 4 | 6 | 10 | Low grade glioma (LGG) | I |
|  |  |  | CSF | 7.5ml | 8 | 5 | 13 |  |  |
| 66 | M | 9 | Blood | 7.5ml | 4 | 4 | 8 | Anaplastic Ependymoma (AEPN) | III |
|  |  |  | CSF | 7.5ml | 6 | 2 | 8 |  |  |
| 67 | M | 4 | Blood | 7.5ml | 4 | 0 | 4 | Medulloblastoma (MB) | IV |
|  |  |  | CSF | 7.5ml | 9 | 2 | 11 |  |  |
| 68 | F | 10 | Blood | 7.5ml | 5 | 7 | 12 | Germ-cell tumor | IV |
|  |  |  | CSF | 7.5ml | 2 | 1 | 3 |  |  |
| 69 | M | 4 | Blood | 7.5ml | 4 | 1 | 5 | Medulloblastoma (MB) | IV |
|  |  |  | CSF | 7.5ml | 4 | 7 | 11 |  |  |
| 70 | F | 3 | Blood | 7.5ml | 0 | 1 | 1 | Medulloblastoma (MB) | IV |
|  |  |  | CSF | 7.5ml | 1 | 1 | 2 |  |  |
| 71 | M | 10 | Blood | 7.5ml | 1 | 1 | 2 | Medulloblastoma (MB) | IV |
|  |  |  | CSF | 7.5ml | 5 | 3 | 8 |  |  |
| 72 | F | 3 | Blood | 7.5ml | 2 | 0 | 2 | Anaplastic Ependymoma (AEPN) | III |
|  |  |  | CSF | 7.5ml | 1 | 0 | 0 |  |  |

**Supplementary Table 3. Gene mution by NGS in CTC and tumor tissue.**

| sample | Gene | Exon position | Nucleotide change | Mutation ratio/copy number |
| --- | --- | --- | --- | --- |
| CSF | CIC | exon20 | c.4783deIT | 9.62% |
|  | MLH1 | exon12 | c.T1151A | 48.68% |
|  | SOX17 | exon2 | c.807＿808AT | 43.10% |
|  | KMT2A | exon13 | c.G4678A | 48.33% |
|  | TMPRSS2 | exon3 | c.T133G | 45.68% |
| Tissue | APC | Exon16 | c.C4621T | 3.18% |
|  | KMT2A | exon13 | c.G4678A | 66.51% |
|  | TMPRSS2 | exon3 | c.T133G | 49.89% |
|  | MSI1 | exon13 | c.G967A | 4.35% |
